# A High‐Fidelity RNA‐Targeting Cas13X Downregulates Connexin43 in Macroglia: A Novel Neuroprotective Strategy for Glaucoma

**DOI:** 10.1002/advs.202415856

**Published:** 2025-06-19

**Authors:** Guoli Zhao, Zhen Li, Ming‐Jie Zhao, Shu‐Ying Li, Qing Xia, Shuoyu Xu, Yu Zhang, Yi Wang, Fang Li, Yu‐Ling Liu, Yun‐Hui Guo, Ruo‐Xi Xu, Han Zhou, Hong Zhou, Wen‐Wen Ding, Yong‐Chen Wang, Yanying Miao, Zhongfeng Wang

**Affiliations:** ^1^ State Key Laboratory of Brain Function and Disorders and MOE Frontiers Center for Brain Science Institutes of Brain Science Fudan University Shanghai 200032 China; ^2^ Eye Institute and Department of Ophthalmology, Eye & ENT Hospital, Fudan University; Key Laboratory of Myopia and Related Eye Diseases, NHC; Key Laboratory of Myopia and Related Eye Diseases Chinese Academy of Medical Sciences Shanghai 200031 China; ^3^ Institute of Neuroscience and Third Affiliated Hospital Zhengzhou University Zhengzhou 450052 China

**Keywords:** Cx43, gene therapy, glaucoma, glia cell, hfCas13X, RGCs

## Abstract

Glaucoma is a neurodegenerative disease characterized by the progressive degeneration of retinal ganglion cells (RGCs) and their axons, ultimately leading to irreversible vision loss. Elevated intraocular pressure (IOP) is one of the significant risk factors in glaucoma; however, neurodegeneration continues even after effective IOP management, underscoring the need for neuroprotective therapies. This study investigates the role of connexin43 (Cx43), which is extensively expressed in retinal macroglia, in regulating microglial activation and optic nerve degeneration in glaucoma. A high‐fidelity CRISPR‐Cas13 (hfCas13X) system is employed to selectively target and knock down Cx43 expression in macroglia. The findings reveal that Cx43‐mediated ATP release through hemichannels exacerbates microglial activation and neuroinflammation, thereby contributing to RGC loss. Notably, in a mouse model of chronic ocular hypertension (COH) glaucoma, knocking down Cx43 in macroglia using the hfCas13X system significantly promoted the survival of RGCs and the integrity of the optic nerve, and improved visual function. The hfCas13X system, which offers high‐fidelity RNA editing with minimal off‐target effects, represents a novel and promising therapeutic strategy for glaucoma, highlighting the potential of gene editing technologies in the management of neurodegenerative diseases.

## Graphical abstract

This study highlights the role of connexin43 (Cx43) in mediating glia activation and neuroinflammation in glaucoma. Using Cx43 knockout mice, it is shown that macroglial cells‐derived Cx43 modulates microglial activation, proliferation, migration, and polarization. Targeting Cx43 with the hfCas13X RNA editing system promoted retinal ganglion cell survival, optic nerve integrity, and improved visual function, offering a promising therapeutic strategy for glaucoma.

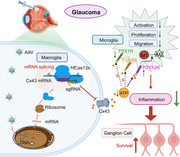

## 1 Introduction

Glaucoma is a neurodegenerative disease characterized by the progressive loss of retinal ganglion cells (RGCs) and their axons, leading to irreversible vision impairment and blindness. Elevated intraocular pressure (IOP) is one of the significant risk factors for glaucoma. Currently, the only proven and widely accepted form of treatment to prevent the progression of glaucoma is lowering IOP. However, even with effective IOP control, some patients' vision continues to deteriorate, suggesting that factors other than elevated IOP contribute to the ongoing loss of RGCs. Therefore, developing neuroprotective therapies for glaucoma is of utmost importance. The mechanisms underlying RGC apoptosis in glaucoma are complex but are generally believed to be related to abnormal interactions between retinal glial cells and RGCs. Retinal inflammatory factors are primarily produced by activated glial cells, leading to changes in the retinal microenvironment and, consequently, inducing damage to RGCs.^[^ ^1^, ^2^ ^]^

Macroglial cells (Müller cells and astrocytes) and microglia maintain a delicate balance and work synergistically to stabilize the retinal microenvironment. Previous studies have shown that in the glaucomatous retina, activated Müller cells release adenosine triphosphate (ATP) through connexin43 (Cx43) hemichannels, which subsequently activates microglia via the nuclear factor of activated T cells (NFAT) and nuclear factor kappa‐B (NF‐κB) signaling pathways acting on P2×7 receptors (P2×7R).^[^ ^3^, ^4^ ^]^ The inflammatory factors released by activated microglia, in turn, upregulate the expression of pro‐inflammatory factors in Müller cells through positive feedback, accelerating retinal neuroinflammation.^[^ ^3^ ^]^ In the retina, astrocytes are mainly distributed in the nerve fiber layer (NFL) and the optic nerve head (ONH).^[^ ^5^ ^]^ In glaucomatous optic neuropathy, the interaction between astrocytes and microglia accelerates optic nerve damage through inflammatory responses.^[^ ^6^, ^7^ ^]^ In acute optic nerve injury, retinal microglia are attracted to the vicinity of dying RGCs and their axons.^[^ ^8^ ^]^ During optic nerve injury, microglia regulate glial remodeling, leading to the significant upregulation of inflammatory marker genes, accelerating optic nerve damage, RGC loss, and thinning of the NFL.^[^ ^9^ ^]^ In the glaucomatous retina, extracellular ATP levels are elevated, and Cx43 channels in astrocytes are one of the primary sources of ATP release.^[^ ^10^ ^]^ Therefore, astrocytes may regulate microglia through a mechanism similar to that of Müller cells in glaucomatous optic nerve injury, thereby contributing to axonal damage in glaucoma. During this process, Cx43 serves as an important regulator of retinal macroglia to microglia communication in glaucoma.

Cx43 is a crucial gap junction protein widely expressed in retinal astrocytes and Müller cells.^[^ ^10^ ^]^ Cx43 regulates the exchange of electrolytes and metabolites between cells by forming hemichannels or complete gap junction channels that connect with other cells. Abnormal activation of Cx43 hemichannels can lead to the pathological release of ATP, triggering inflammatory responses, which is particularly significant in neurodegenerative diseases like glaucoma.^[^ ^11^ ^]^ While Cx43‐mediated gap junction communication may provide neuroprotective effects in the early stages of glaucoma, it can also facilitate the spread of pathological changes when metabolic resources are depleted.^[^ ^12^, ^13^ ^]^ In glaucoma, the increase in activity of Cx43 channels may exacerbate the over‐activation of glial cells, promoting the release of pro‐inflammatory cytokines that further contribute to neuronal damage and optic nerve degeneration.^[^ ^14^ ^]^ Therefore, Cx43 serves not only as a key regulator of inflammation and neurodegeneration in glaucoma but also as a potential therapeutic target.

Gene editing techniques have shown great promise in treating retinal diseases.^[^ ^15^, ^16^ ^]^ Knocking down Cx43 expression through gene editing could be an effective strategy to modulate glial cell‐mediated inflammation and treat glaucoma. Specifically, using RNA editing tools like the hfCas13X system can effectively suppress Cx43 expression. The hfCas13X system represents a significant advancement in RNA editing techniques, offering a high‐fidelity Cas13 variant with minimal off‐target effects, addressing the limitations of the CasRx system. Unlike traditional DNA‐targeting CRISPR systems, hfCas13X specifically targets RNA, enabling precise and potent knockdown of target genes without altering the genome, making the editing process reversible and safe.^[^ ^17^ ^]^ The hfCas13X system holds great promise for the treatment of ocular diseases.^[^ ^18^, ^19^ ^]^

In this study, we explored the role of Cx43 expression in astrocytes in regulating microglial activation and optic nerve degeneration in glaucoma. Utilizing the hfCas13X system, we precisely modulated Cx43 gene expression to influence microglial behavior, achieving significant neuroprotection and improved visual function. Our findings highlight the substantial potential of gene editing techniques targeting Cx43 for neuroprotection in glaucoma, paving the way for the development of more effective therapeutic strategies.

## 2 Results

### 2.1 Astrocytes‐Induced Microglial Activation, Proliferation, and Migration in the ONH

**Figure 1:**
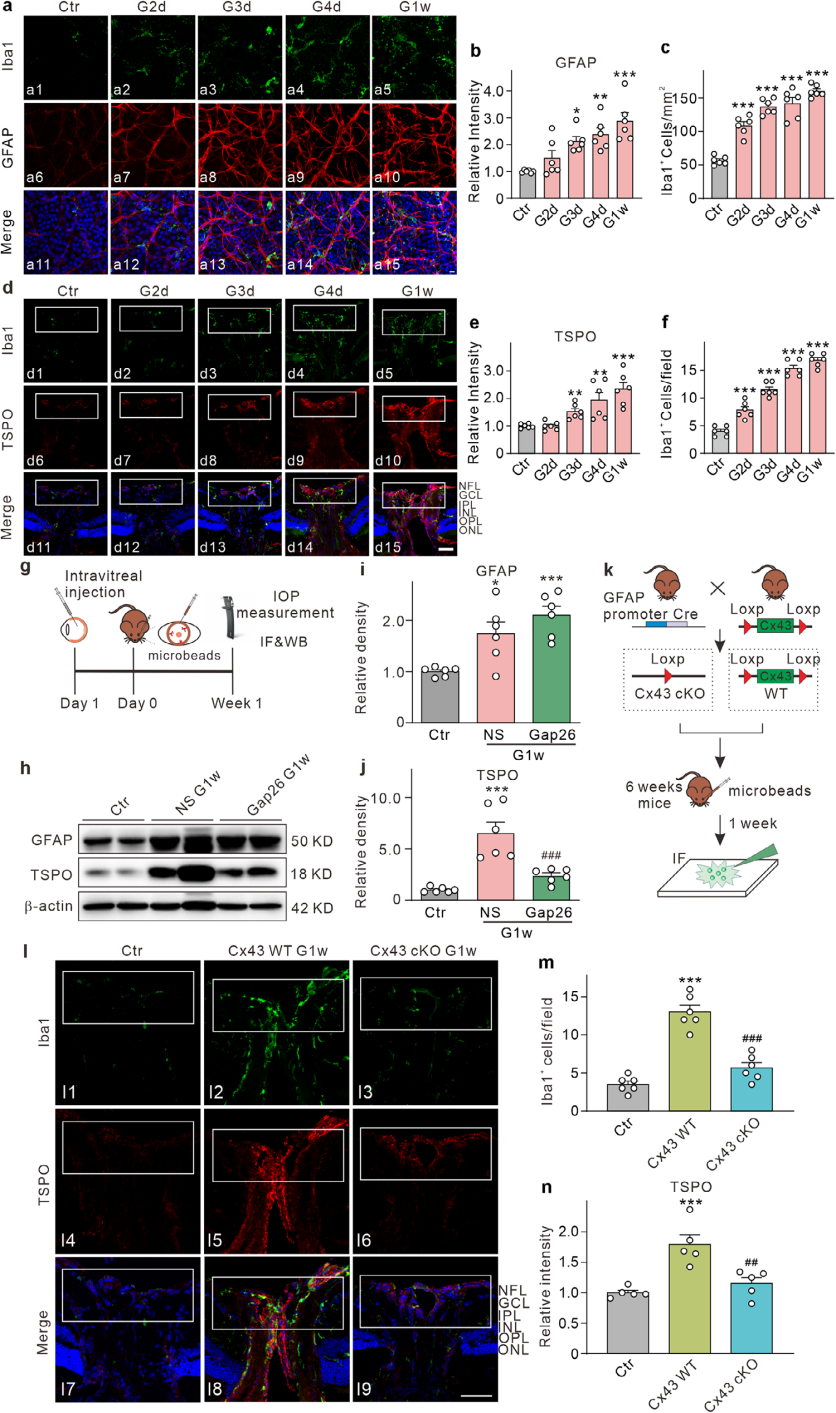
Cx43 in astrocytes mediated influence on microglial activation, proliferation, and/or migration in COH mice. a) Representative immunofluorescence images are taken from whole flat‐mounted retinas staining with GFAP and Iba1 in the control group (Ctr, a1, a6, a11) and 2nd (G2d, a2, a7, a12), 3rd (G3d, a3, a8, a13), 4th day (G4d, a4, a9, a14) and 1w (G1w, a5, a10, a15) after IOP elevation in COH mice. Scale bar: 10 µm for all images. b) Quantification of the relative average intensity of GFAP fluorescence in whole flat‐mounted retinas from the control (Ctr) and experimental groups (G2d, G3d, G4d, and G1w) in COH mice. c) Quantification of Iba1^+^ microglial cells in whole flat‐mounted retinas from the control (Ctr) and experimental groups (G2d, G3d, G4d, and G1w) in COH mice. Data (for b and c panels) were collected from eight regions of the retina: four from the central region (800 µm from the ONH) and four from the peripheral region (1,600 µm from the ONH) at angles of 0, 90, 180 and 270° of the retina, respectively. The area of each region is 0.25 mm^2^. n = 6 mice. ^*^ *P* < 0.05, ^**^ *P* < 0.01, and ^***^ *P* < 0.001 versus Ctr. One‐way ANOVA with Tukey's multiple comparisons test. d) Double immunofluorescence staining showing the expression of Iba1 (d1–d5) and TSPO (d6–d10) in vertical sections of the ONH taken from control (Ctr), and COH mice at G2d, G3d, G4d, and G1w, respectively. Scale bar: 50 µm for all images. e) The bar chart shows the changes in the average number of Iba1‐labeled microglia in the white rectangular area in panel d. n = 6 mice. ^**^ *P* < 0.01 and ^***^ *P* < 0.001 versus Ctr. One‐way ANOVA with Tukey's multiple comparisons test. f) A bar chart showing the relative average intensity of TSPO fluorescence signals in the white rectangular area is shown in panel d. n = 6 mice. ^*^ *P* < 0.05, ^***^ *P* < 0.001 versus Ctr. One‐way ANOVA with Tukey's multiple comparisons test. g) Diagram of the experimental procedure for establishing a COH model after injection of the drug. h–j) Representative immunoblots (h) and quantification of GFAP (i) and TSPO (j) protein levels in Ctr, NS G1w and Gap26 G1w groups. All data are normalized to their corresponding β‐actin and then to the Ctr. n = 6 mice. ^*^ *P* < 0.05 and ^***^ *P* < 0.001 versus Ctr; ^###^ *P* < 0.001 versus NS G1w. One‐way ANOVA with Tukey's multiple comparisons test. k) Mating reproduction diagram of Cx43 cKO transgenic mice and the following experimental procedure. l) Double immunofluorescence staining of Iba1 and TSPO in vertical sections of the ONH in Ctr (l1, l4, l7), Cx43 WT (l2, l5, l8) and Cx43 cKO (l3, l6, l9) COH mice. Scale bar: 50 µm for all images. m) Quantification of Iba1 labeled cells in the white rectangular area in panel l. n) Bar chart showing the relative average intensity of TSPO fluorescence signals in the white rectangular area in panel j. n = 5–6 mice. ^***^ *P* < 0.001 versus Ctr, ^##^ *P* < 0.01, ^###^ *P* < 0.001 versus Cx43 WT. One‐way ANOVA with Tukey's multiple comparisons test (for m and n panels). NFL, nerve fiber layer; GCL, ganglion cell layer; IPL, inner plexiform layer; INL, inner nuclear layer, OPL; outer plexiform layer; ONL, outer nuclear layer.

Previous studies have demonstrated that activated microglia migrate to the NFL/ganglion cell layer (GCL).^[^ ^3^, ^4^ ^]^ We tested whether activated astrocytes may influence microglial activation, proliferation, and/or migration in chronic ocular hypertension (COH) experimental glaucoma mice (Figure S1, Supporting Information). Retinal whole‐mount staining was conducted using glial fibrillary acidic protein (GFAP) and Iba1 (a marker of microglia) antibodies to evaluate astrocyte activation and the change of microglia in the NFL during the early stages of IOP elevation in COH mice. As illustrated in **Figure** **1**a, GFAP fluorescence intensity increased from the second day after IOP elevation (G2d) until 1w (G1w) compared to the control group (Figure 1b), indicating significant astrocyte activation, characterized by cell body enlargement and increased branching. Accompanying astrocyte activation, the number of Iba1‐positive microglia was significantly increased from G2d to G1w (Figure 1a,c). Morphologically, microglia exhibited an amoeboid shape, characterized by enlarged cell bodies in COH retinas, indicative of microglia activation (Figure 1a,a2–a5).

Microglia in the ONH and retina were stained using Iba1 and anti‐translocator protein (TSPO) (a marker of activated microglia).^[^ ^3^ ^]^ The results indicated an increase in the number of Iba1‐positive microglia in the NFL of the ONH starting from G2d. Additionally, TSPO fluorescence intensity exhibited a slight increase three days after IOP elevation (G3d) compared to the control group, suggesting early microglial activation (Figure 1d–f). The retinal staining results were consistent with those observed in the ONH (Figure S2g, Supporting Information). These findings imply that astrocytes became activated, coinciding with the proliferation and/or migration of microglia.

Since the effects of activated astrocytes on microglia have been observed before G1w in the ONH of COH mice, mechanism analyses were done within 1 week after IOP elevation in COH mice. To further elucidate the role of astrocytes in microglial activation, we depleted retinal astrocytes through intravitreal injection of L‐2‐aminoadipic (LAA), a glial toxin that selectively impairs astrocyte function.^[^ ^20^ ^]^ As illustrated in Figure S3 (Supporting Information), the number of GFAP‐positive astrocytes significantly decreased three days post‐LAA injection, with a gradual recovery observed by day 8. COH model was established one day after LAA pre‐injection. LAA treatment significantly reduced the number of microglia in the NFL of the ONH at G4d and G1w compared to the control group (normal saline, NS), indicating that astrocyte depletion attenuated microglial proliferation and/or migration. Furthermore, TSPO fluorescence intensity was significantly diminished at G4d following LAA pre‐injection. Microglia displayed classic amoeboid morphology at G4d and G1w, characterized by enlarged cell bodies and reduced branching. Depletion of astrocytes through LAA treatment resulted in a significant reduction in the number of activated microglia (Figure S2a–c, Supporting Information). Examination of retinal sections further demonstrated that LAA pre‐injection similarly decreased microglial populations within the NFL (Figure S2h, Supporting Information). These findings indicate that activated astrocytes are essential in modulating microglial activation, proliferation, and/or migration in COH retinas.

### 2.2 ATP Releasing via Cx43 Hemichannels in Astrocytes Influences Microglial Activation, Proliferation, and Migration

Astrocytes are known to release ATP through Cx43 hemichannels, which activate purinergic receptors, facilitating communication between astrocytes and microglia.^[^ ^6^, ^21^, ^22^ ^]^ Given the significant influence of astrocytes on microglial activation, proliferation, and/or migration observed earlier, we hypothesized that ATP release via Cx43 hemichannels in astrocytes could trigger these microglial changes. To investigate this hypothesis, we pre‐injected the Cx43 channel blocker Gap26 prior to establishing the COH model and evaluated its effects on microglial activation, proliferation, and migration. As illustrated in Figure S2d–f (Supporting Information), Gap26 pre‐injection resulted in a significant reduction in the number of microglia within the NFL of the ONH at G1w. Gap26 pre‐injection notably diminished IOP elevation‐induced increase in TSPO fluorescence intensity at G1w in COH mice. The changes in microglial numbers observed in retinal sections were consistent with those noted in the ONH (Figure S2i, Supporting Information). Furthermore, Western blot analysis revealed a significant upregulation of GFAP and TSPO expression at G1w. While the inhibition of Cx43 hemichannels did not significantly alter GFAP expression, it markedly reduced TSPO expression (Figure 1h–j). Collectively, these findings suggest that blocking Cx43 channels inhibits microglial activation, proliferation, and migration, potentially mediated by ATP release through Cx43 hemichannels.

Given that Cx43 is expressed in astrocytes, Müller cells, and microglia, we conducted further experiments using conditional astrocyte‐specific Cx43 conditional knockout (CX43 cKO) mice to validate the astrocyte‐specific regulation of microglia. Cre expression was found to co‐localize with GFAP in the retinas of Cx43 cKO mice, indicating that Cre was expressed in astrocyte nuclei; however, not all astrocytes exhibited Cre expression, leading to incomplete knockout efficiency (Figure S4a, Supporting Information). Furthermore, in the COH retina at G1w, Cre expression was also observed in Müller cells (Figure S4b, Supporting Information). Western blot analysis corroborated a significant reduction in Cx43 levels in the retinas of Cx43 cKO mice (Figure S4c,d, Supporting Information). Double immunofluorescence staining for GFAP and Cx43 revealed abundant Cx43 expression in GFAP‐positive cells from Cx43 wild‐type (WT) mice, whereas Cx43 expression was nearly absent in Cx43 cKO mice (Figure S4g, Supporting Information). Additionally, Cx43 cKO did not affect the expression of GFAP, as evidenced by both immunofluorescence and Western blot analyses (Figure S4e,f,h–j, Supporting Information). Subsequent experiments utilizing the COH model in Cx43 cKO mice demonstrated a significant decrease in Iba1‐positive microglia in the NFL within the ONH at G1w compared to controls, alongside a notable reduction in TSPO fluorescence intensity (Figure 1l–n). These findings suggest that the conditional astrocyte‐specific knockout of Cx43 inhibits microglial activation, proliferation, and/or migration in COH mice. Similar results were observed in retinal vertical sections (Figure S2j, Supporting Information).

**Figure 2:**
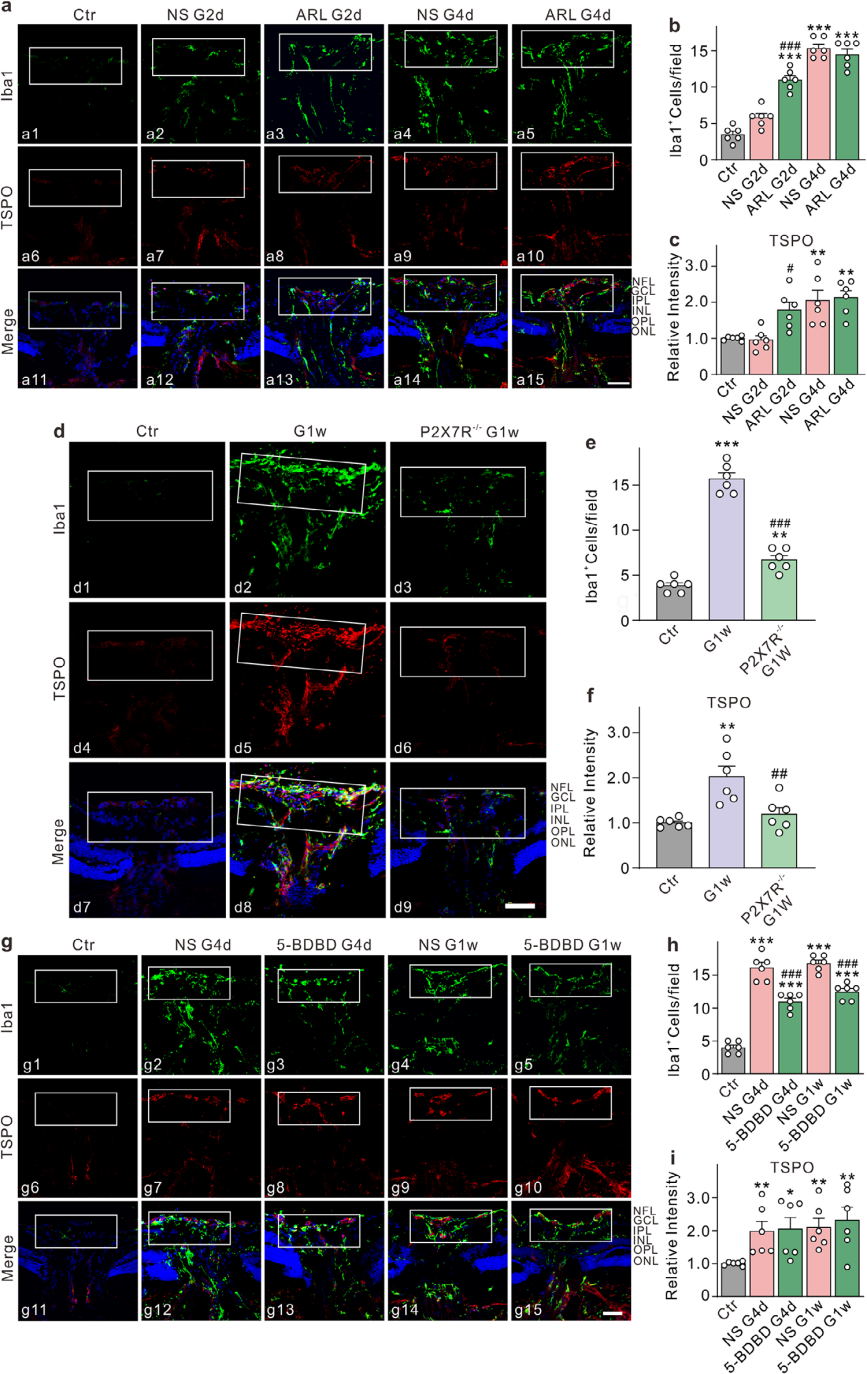
P2×7R/P2×4R‐mediated effects on microglia activation, proliferation, and/or migration in COH mice. a) Double immunofluorescence staining of Iba1 (a1–a5) and TSPO (a6–a10) in vertical sections of the ONH in Ctr, NS, and ARL67156 (ARL) injected COH mice at G2d, G4d, respectively. Scale bar: 50 µm for all images. b) Quantification of Iba1 labeled cells in the white rectangular area in panel a. c) Bar chart showing the relative average intensity of TSPO fluorescence signals in the white rectangular area in panel a. n = 6 mice. ^**^ *P* < 0.01 and ^***^ *P* < 0.001 versus Ctr. ^#^ *P* < 0.05 and ^###^ *P* < 0.001 versus NS G2d. d) Double immunofluorescence images showing the expression of Iba1 (d1–d3) and TSPO (d4–d6) in vertical slices of the ONH in Ctr, G1w, and *P2×7R* ^−/−^ G1w. Scale bar: 50 µm for all images. e) Bar chart showing the average number of Iba1 labeled microglia in the white rectangular area in panel d. f) A Bar chart showing the relative average intensity of TSPO fluorescence signals in the white rectangular area is shown in panel d. n = 6 mice. ^**^ *P* < 0.01, ^***^ *P* < 0.001 versus Ctr. ^##^ *P* < 0.01, ^###^ *P* < 0.001 versus G1w.One‐way ANOVA with Tukey's multiple comparisons test for b, c, e, f. g) Double immunofluorescence images showing the expression of Iba1 (g1–g5) and TSPO (g6–g10) in vertical slices of the ONH in Ctr, NS and 5‐BDBD injected COH mice at G4d and G1w, respectively. Scale bar: 50 µm for all images. h) Bar chart showing the average number of Iba1 labeled microglia in the white rectangular area in panel g. n = 6 mice. ^***^ *P* < 0.001 versus Ctr. ^###^ *P* < 0.001 versus NS G4d or NS G1w. One‐way ANOVA with Tukey's multiple comparisons test and unpaired *t‐*test. i) The bar chart shows the relative average intensity of TSPO fluorescence signals in the white rectangular area in panel g. n = 6 mice. Unpaired *t*‐test with comparisons between each experimental group and Ctr. ^*^ *P* < 0.05, ^**^ *P* < 0.01 versus Ctr.

To further investigate whether ATP released through Cx43 channels from astrocytes is responsible for the regulation of microglia, we pre‐injected an ATPase inhibitor, ARL67156, into the vitreous cavity prior to establishing the COH model. The results indicated that the inhibition of ATP degradation significantly increased the number of microglia in the ONH at G2d; however, by G4d, the inhibition of ATP degradation no longer resulted in an increased microglial count compared to the NS G4d group (**Figure** **2**a,b). It suggests that by G4d, the extracellular ATP concentration is already sufficiently elevated to promote microglial proliferation and migration, thereby rendering any further increase in ATP concentration less effective. Additionally, TSPO fluorescence intensity exhibited a slight increase at G2d following ATP degradation inhibition (Figure 2a,c). Staining of retinal sections for Iba1 and TSPO yielded results that were consistent with those observed in the ONH (Figure S5a, Supporting Information).

### 2.3 ATP Regulates Microglial Activation, Proliferation, and Migration via P2×7R/P2×4R

ATP serves as a crucial regulator of microglial activity, functioning as an endogenous ligand for the P2 purinergic receptor family. Among these receptors, P2×7R and P2×4R are the predominant subtypes expressed in microglia.^[^ ^23^, ^24^ ^]^ Prior research has established that in COH mouse retinas, activated Müller cells facilitate microglial activation and proliferation through ATP/P2×7R interactions while also inducing microglial migration via P2×4R/P2×7R pathways.^[^ ^3^, ^4^ ^]^ In this study, we investigated the mechanism by which astrocyte‐derived ATP, released through Cx43 hemichannels, influences microglial responses at the ONH. We initially employed *P2×7R^−/−^* transgenic mice to assess the role of the P2×7R in microglial behavior. Following the establishment of the COH model, we noted a significant reduction in the number of microglia within the NFL in P2×7R knockout mice compared to the control group at G1w. Furthermore, microglia in *P2×7R^−/−^* mice displayed smaller cell bodies with elongated branches, and TSPO fluorescence intensity was markedly diminished relative to the G1w group (Figure 2d–f; Figure S5b, Supporting Information). These findings indicate that the P2×7R mediates microglial activation and changes in microglial numbers in COH mice, likely due to microglial proliferation and migration.

To further investigate the role of the P2×4R, we pre‐injected the P2×4R blocker 5‐BDBD before establishing the COH model and collected samples at G4d and G1w. Results showed that P2×4R inhibition significantly reduced the number of microglia in the NFL at G4d and G1w, though the reduction was less pronounced than that observed with P2×7R knockout. TSPO fluorescence staining indicated that P2×4R inhibition did not significantly affect microglial activation (Figure 2g–i; Figure S5c, Supporting Information). The results described above suggest that P2×4R inhibition primarily reduces microglial migration rather than activation. Additionally, LAA, Gap 26, and ARL67156 had no significant effects on microglia in normal mice (Figure S5d–f, Supporting Information), and the numbers of microglia in *P2×7R^−/−^* mice or in 5‐BDBD injected mice did not show significant change.^[^ ^4^ ^]^

### 2.4 Impact and Mechanism of Cx43 Hemichannel‐Mediated ATP Release on Microglial Pro‐Inflammatory and Anti‐Inflammatory Phenotypes

Microglia exhibit heightened sensitivity to alterations in the retinal environment, dynamically transitioning between pro‐inflammatory M1‐like and anti‐inflammatory M2‐like phenotypes in response to neurodegenerative diseases or trauma. M1‐like microglia release pro‐inflammatory factors that intensify inflammation and contribute to neuronal damage, whereas M2‐like microglia secrete neuroprotective factors that facilitate disease resolution.^[^ ^25^, ^26^ ^]^ To evaluate the dynamic phenotypic changes of microglia in the ONH of COH mice during a period of G4d to G4w, we conducted immunostaining using the M1 marker CD86 and the M2 marker CD206. As illustrated in Figure S6 (Supporting Information), the population of Iba1/CD206 positive microglia exhibited a significant increase from G4d to G3w, peaking at G2w before gradually declining. The number of Iba1/CD86 double‐positive microglia rose at G4d and continued to escalate until G4w (Figure S6a–d, Supporting Information). These findings indicate that M2‐like microglia primarily increase during the early stages of IOP elevation, while M1‐like microglia progressively rise and sustain elevated levels post‐G2w. This suggests that subsequent microglial activation could exacerbate optic nerve damage through inflammatory processes. Notably, the microglial phenotype in the NFL mirrored that observed in the optic nerve papilla (Figure S6e–h, Supporting Information).

**Figure 3:**
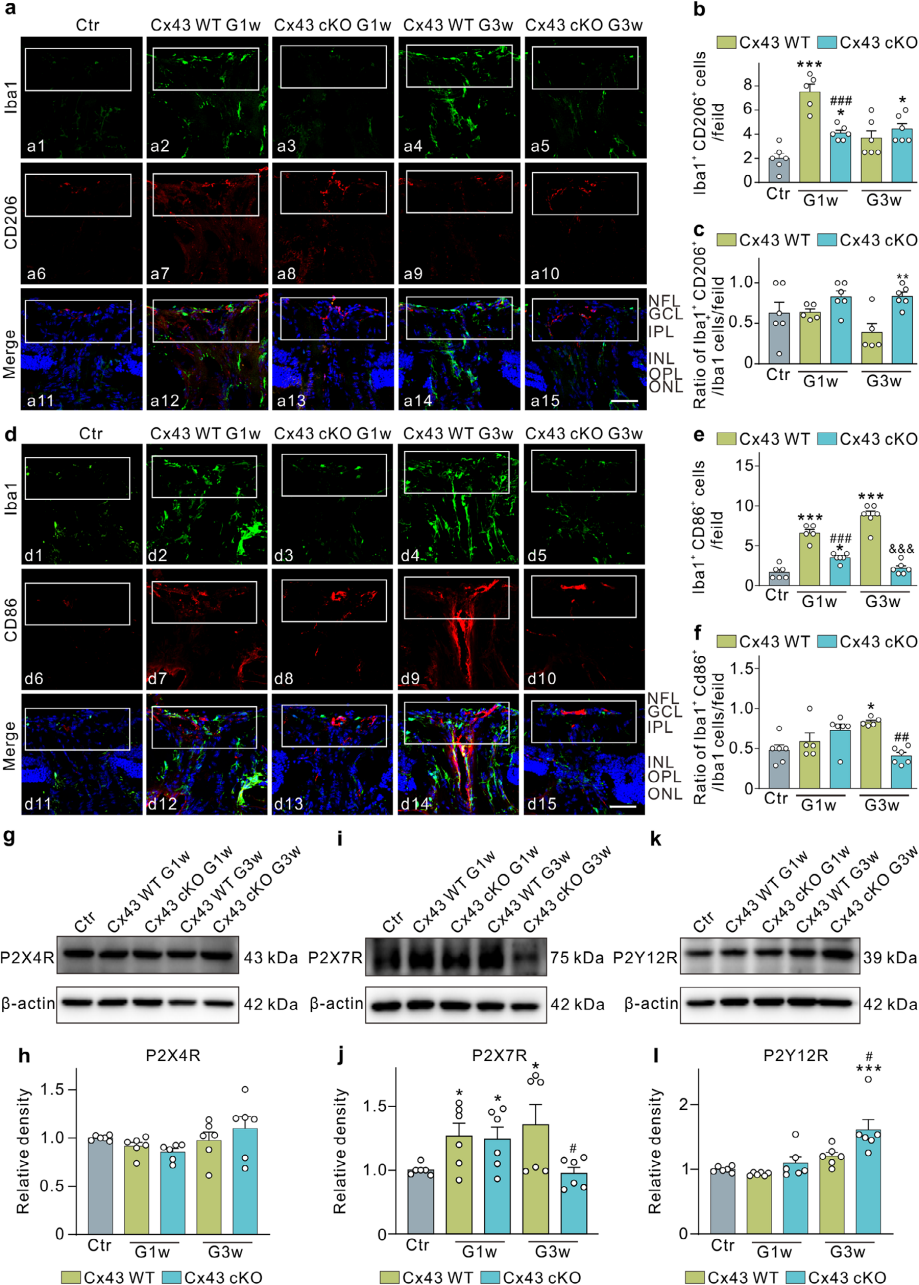
Effects of Cx43 conditional knockout (Cx43 cKO) in astrocytes on the ratio of pro‐inflammatory M1‐like and anti‐inflammatory M2‐like microglia in COH mice. a) Representative images of immunostaining Iba1 (a1–a5) and CD206 (a6–a10) in vertical slices of the ONH in Ctr, Cx43 WT, and Cx43 cKO COH mice at G1w and G3w. Scale bar: 50 µm for all images. b) Bar charts showing the average number of Iba1 and CD206 labeled microglia in the white rectangular area in panel a. n = 5–6 mice. ^*^ *P* < 0.05, ^***^ *P* < 0.001 versus Ctr; ^###^ *P* < 0.001 vs. Cx43 WT G1w. One‐way ANOVA with Tukey's multiple comparisons test. c) Bar charts showing the ratios of the number of M2‐like microglia and total microglia. n = 5–6 mice. ^**^ *P* < 0.01 versus Cx43 WT G3w. Unpaired *t*‐test. d) Representative images of immunostaining Iba1 (d1–d5) and CD86 (d6–d10) in vertical slices of the ONH in Ctr, Cx43 WT, and Cx43 cKO COH mice at G1w and G3w. Scale bar: 50 µm for all images. e) Bar charts showing the average number of Iba1 and CD86 labeled microglia in the white rectangular area in panel d. n = 5–6 mice. ^***^ *P* < 0.001 versus Ctr; ^###^ *P* < 0.001 versus Cx43 WT G1w; ^&&&^ *P* < 0.001 versus Cx43 WT G3w. One‐way ANOVA with Tukey's multiple comparisons test. f) Bar charts showing the ratios of the number of M1‐like microglia and total microglia. n = 5–6 mice. ^*^ *P* < 0.05 versus Ctr; ^##^ *P* < 0.01 versus Cx43 WT G3w. A non‐parametric Kruskal‐Wallis ANOVA test was acquired to confirm statistical significance. g–l), Representative images (g,i,k) and quantification (h,j,l) of P2×4R, P2×7R and P2Y12R protein levels in Ctr, Cx43 WT and Cx43 cKO COH mice at G1w and G3w. n = 6 mice. ^*^ *P* < 0.05, ^***^ *P* < 0.001 versus Ctr; ^#^ *P* < 0.05 versus Cx43 WT G3w. One‐way ANOVA with Tukey's multiple comparisons test for h and i. Unpaired *t*‐test with comparisons between each experimental group and Ctr, between Cx43 WT G3w and Cx43 cKO G3w for j.

In the oxygen‐glucose deprivation/reperfusion model, inhibiting the opening of Cx43 hemichannels and the release of ATP in astrocytes promotes a shift in activated microglia from an M1‐like to an M2‐like phenotype, thereby providing neuroprotection.^[^ ^11^ ^]^ Based on these findings, we hypothesized that in the COH model, ATP released from astrocytes through Cx43 hemichannels influences the phenotypic changes of microglia. To investigate this hypothesis, we established the COH model in Cx43 cKO mice and assessed microglial populations in ONH sections using CD206 and CD86 staining. Based on the dynamic changes of M1‐/M2‐like microglia in the COH retina, two time points (G1w and G3w) were chosen. As shown in **Figure** **3**a,b, the number of Iba1/CD206 double‐positive microglia in Cx43 cKO mice at G1w was significantly reduced compared to that in Cx43 WT mice. At G3w, the number of Iba1/CD206 double‐positive microglia exhibited a slight increase relative to Cx43 WT mice. Our analysis indicated that the astrocytic Cx43 cKO significantly decreased the number of Iba1/CD206 double‐positive microglia at G1w but had no significant effect at G3w, which was inconsistent with our expectations. Importantly, the overall reduction in microglial numbers due to Cx43 knockout likely contributed to the observed decrease in M2‐like microglia. Consequently, we calculated the ratio of Iba1/CD206 double‐positive microglia to total Iba1‐positive microglia. The results, presented in Figure 3c, demonstrate that the conditional knockout of astrocytic Cx43 did not significantly alter the proportion of M2‐like microglia at G1w, but it significantly increased this proportion at G3w compared to Cx43 WT mice.

Next, we investigated the impact of astrocyte Cx43 cKO on M1‐like microglia. As illustrated in Figure 3d,e, the number of Iba1/CD86 double‐positive microglia in Cx43 cKO mice at G1w was significantly lower compared to Cx43 WT mice, and this reduction persisted at G3w. The analysis of the ratio of Iba1/CD86 double‐positive microglia to total Iba1‐positive microglia, as shown in Figure 3f, indicated that Cx43 knockout did not significantly alter the proportion of M1‐like microglia at G1w, but it did lead to a significant reduction at G3w compared to Cx43 WT mice.

In multiple sclerosis, the P2×7R is linked to the pro‐inflammatory phenotype of microglia, whereas the P2Y12R is associated with the anti‐inflammatory phenotype.^[^ ^27^, ^28^ ^]^ The P2Y12R is predominantly expressed in M2‐like microglia, with lower expression levels in M1‐like microglia.^[^ ^29^, ^30^ ^]^ To further investigate the mechanism by which astrocytes regulate microglial phenotypes via Cx43 hemichannels, we performed Western blot analysis to assess the expression of P2×4R, P2×7R, and P2Y12R. At G1w, no significant changes in the expression of P2×4R and P2Y12R were detected in Cx43 cKO mice compared to Cx43 WT mice, while P2×7R expression was increased. However, at G3w, we observed a significant reduction in P2×7R expression alongside a significant increase in P2Y12R expression (Figure 3g–l). These findings suggest that conditional knockout of astrocyte Cx43 may facilitate the transition of microglia to an M2‐like phenotype by activating the P2Y12R while concurrently diminishing M1‐like microglia through the attenuation of ATP stimulation on the P2×7R.

### 2.5 Conditional Knockout of Astrocyte Cx43 Alleviates Inflammation and Protects Visual Function

**Figure 4:**
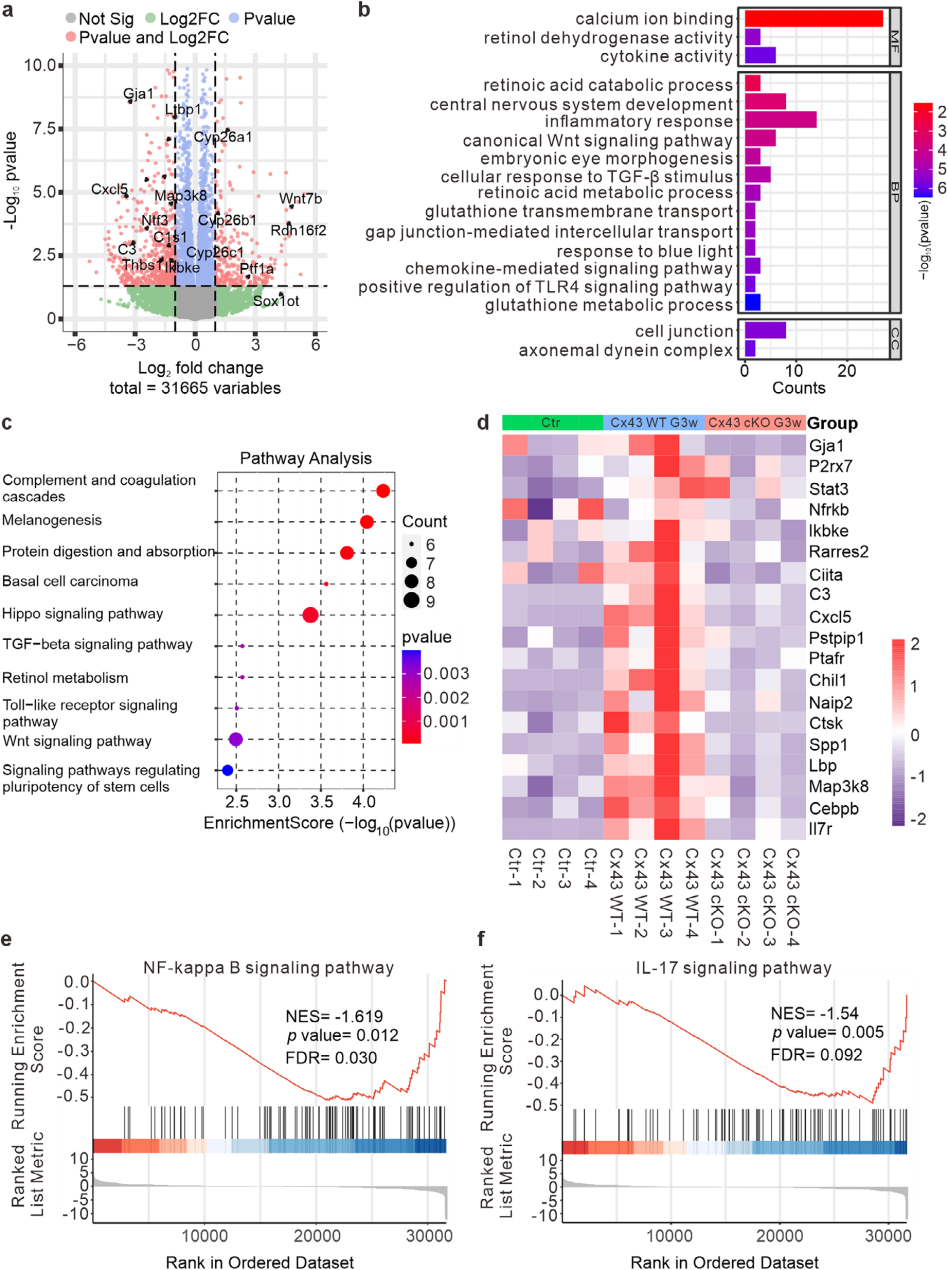
Cx43 conditional knockout (cKO) in astrocytes alleviates inflammation and protects visual function in COH mice. a) Genes volcano map showing differential gene expression between Cx43 WT group and Cx43 cKO group at G3w. n = 4. b) GO enrichment pathways of Cx43 cKO group compared to Cx43 WT group. c) KEGG enrichment of Cx43 cKO group compared to Cx43 WT group (top 10). d) Heatmap showing the differentially expressed genes of Ctr, Cx43 WT, and Cx43 cKO group. e,f) The GSEA analysis of KEGG pathway enrichment between the Cx43 cKO group compared to the Cx43 WT group.

The above results showed that inhibition of astrocytic Cx43 significantly increased the number of M2‐like microglia and reduced the number of M1‐like microglia at G3w but not at G1w. Thus, we chose the G3w time point for evaluating neuroinflammation and RGC function. To further investigate the neuroprotective mechanisms of conditional astrocyte Cx43 knockout in glaucoma, we performed RNA‐seq analysis on retinal tissues from WT, Cx43 WT, and Cx43 cKO COH mice at G3w. Volcano plots of differential gene expression revealed that genes related to neuroregeneration and visual protection, such as *Wnt7b*, *Rdh16f2*, and *Cyp26*, were upregulated in the Cx43 cKO group compared to the Cx43 WT group (**Figure** **4**a). These genes are pivotal in pathways that promote neuronal health and visual function. Specifically, *Wnt7b* is implicated in the Wnt signaling pathway, which is essential for regulating cell fate, proliferation, and survival within the central nervous system.^[^ ^31^ ^]^ *Rdh16f2* is linked to retinoid metabolism, which is vital for preserving photoreceptor function and overall retinal health, whereas *Cyp26* is responsible for regulating retinoic acid levels, which are crucial for eye development and function.^[^ ^32^, ^33^ ^]^ In contrast, genes related to immune response and inflammation, such as *Ikbke*, *Cxcl5*, and *C3*, exhibited downregulation.^[^ ^34^, ^35^, ^36^ ^]^

Gene ontology (GO) and KEGG enrichment analyses provided further insights into the effects of Cx43 knockout. The analyses indicated that astrocyte Cx43 cKO mitigated immune and inflammation‐related pathways, such as “inflammatory response,” “chemokine‐mediated signaling pathway,” and “Toll‐like receptor signaling pathway.” These pathways are critical in initiating and sustaining inflammatory responses that can lead to neurodegeneration in glaucoma. The reduction in activity within these pathways suggests that Cx43 cKO effectively dampens the inflammatory environment within the retina. Conversely, Cx43 cKO appeared to modulate neuroprotective and vision‐related pathways, including “Wnt signaling pathway,” “central nervous system development,” “retinol metabolism,” “embryonic eye morphogenesis,” and “response to blue light.” The modulation of retinol metabolism and response to blue light indicates potential protective effects on photoreceptors and overall retinal health, which are critical in preserving visual function in glaucoma (Figure 4b,c).

Heatmap analysis confirmed the effectiveness of the gene knockout by demonstrating the downregulation of *gja1*, the gene encoding Cx43, alongside several pro‐inflammatory markers, including *p2×7r*, *stat3*, *nfrkb*, and *ikbke*. This downregulation of *p2×7r*, *stat3*, *nfrkb*, and *ikbke* further indicates that the Cx43 cKO in astrocytes reduces the expression of P2×7R and inhibits inflammation‐related pathways, such as the NF‐κB signaling pathway. The diminished expression of inflammatory‐related genes, including *rarres2*, *ciita*, *c3*, *cxcl5*, *pstpip1*, *chil1*, *naip2*, *ctsk*, *spp1*, *lbp*, *map3k8*, *cebpb*, and *il7*, strongly suggests that Cx43 cKO results in a broad attenuation of inflammatory responses in the retina (Figure 4d). These downregulated genes play pivotal roles in various inflammatory pathways and contribute to the pathogenesis of glaucoma. For instance, Rarres2 (retinoic acid receptor responder 2) is involved in regulating immune responses and has been linked to the modulation of macrophage activity, which can affect the inflammatory environment within the retina.^[^ ^37^ ^]^ Ciita (class II major histocompatibility complex transactivator) is a key regulator of MHC class II gene expression, and the reduction in ciita expression suggests a decrease in immune cell infiltration.^[^ ^38^ ^]^ C3 (complement component 3) and cfb (complement factor B) are central components of the complement cascade, which, when over‐activated, can lead to neuroinflammation and retinal damage.^[^ ^39^ ^]^ Additional downregulated genes, such as *ctsk* (cathepsin K) and *spp1* (secreted phosphoprotein 1/osteopontin), are also involved in tissue remodeling and inflammation.^[^ ^40^, ^41^ ^]^ The broad downregulation of these factors indicates a suppression of inflammatory gene expression, which may contribute to the neuroprotective effects observed in Cx43 cKO mice.

Gene set enrichment analysis (GSEA) enrichment analysis further confirmed that Cx43 cKO in astrocytes modulates inflammation‐related pathways, including the “NF‐kappa B signaling pathway” and “IL‐17 signaling pathway” (Figure 4e,f). These results suggest that Cx43 cKO in astrocytes may protect visual pathways by inhibiting inflammatory responses and promoting neuroprotective mechanisms.

### 2.6 Effects of Cx43 cKO in Astrocyte on Optic Nerve Fiber Damage and Axonal Transport

**Figure 5:**
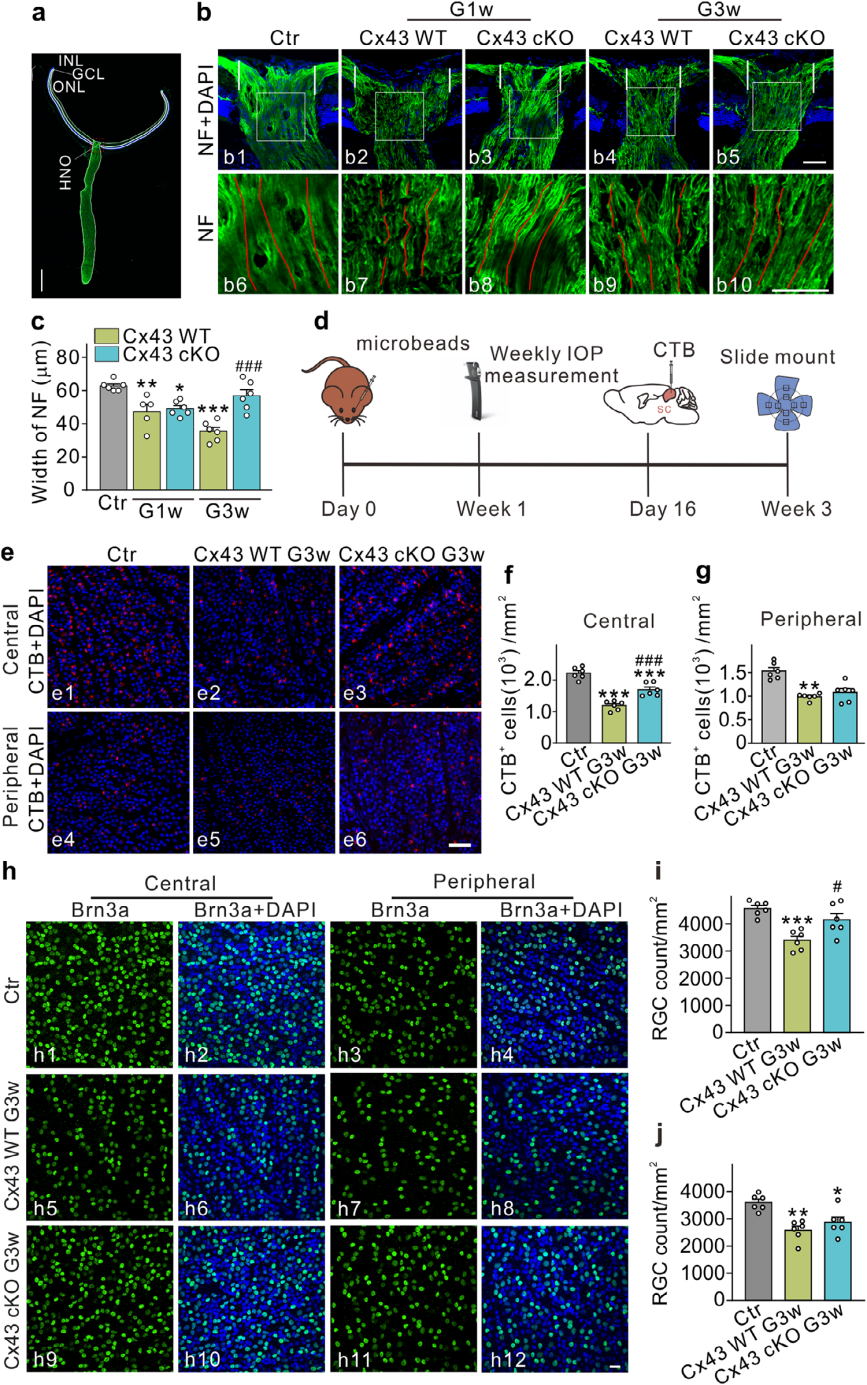
Effects of Cx43 conditional knockout (cKO) in astrocytes on nerve fiber injury and axoplasm transport in COH mice. a) Diagrammatic sketch displaying the field of object image taken from the vertical slices of the optic nerve. Scale bar: 500 µm. b) Representative micrographs showing the immunostaining of neurofilament protein (NF) in the vertical slices of the ONH in Ctr, Cx43 WT, and Cx43 cKO COH mice at G1w and G3w. Scale bar: 50 µm for all images. b6–b10 are enlarged images of the white rectangular area in b1–b5, respectively. The red lines highlight the continuity of the neurofilament fibers. c) Bar charts showing the average width of the nerve fibers marked by the white line in panel b. n = 5–6 mice. ^*^ *P* < 0.05, ^**^ *P* < 0.01, ^***^ *P* < 0.001 versus Ctr; ^###^ *P* < 0.001 versus Cx43 WT G3w. One‐way ANOVA with Tukey's multiple comparisons test. d) Schematic diagram showing the experimental strategy of CTB retrogradely labeled RGCs. Four fields were randomly selected from the center (≈0.8 mm from the ONH) and the peripheral (≈1.6 mm from the ONH), respectively. e) Representative images of CTB retrogradely labeled RGCs in the central (e1–e3) and peripheral (e4–e6) regions taken from whole flat‐mounted retinas in Ctr, Cx43 WT, and Cx43 cKO COH mice at G3w. Scale bar: 50 µm for all images. f,g) Quantification of CTB‐positive cells in the central (f) and peripheral (g) regions under different conditions as shown in panel e. n = 6. ^**^ *P* < 0.01, ^***^ *P* < 0.001 versus Ctr; ^###^ *P* < 0.001 versus Cx43 WT G3w. One‐way ANOVA with Tukey's multiple comparisons test. h) Representative micrographs of Brn3a‐positive RGCs in the central and peripheral regions of the retina in Ctr, Cx43 WT G3w, and Cx43 cKO G3w mice. Scale bar: 20 µm for all images. i,j) Quantification of Brn3a‐positive RGCs in central and peripheral retinal regions, respectively, with the following conditions: Ctr, Cx43 WT G3w, and Cx43 cKO G3w. n = 6. ^*^ *P* < 0.05, ^**^ *P* < 0.01, ^***^ *P* < 0.001 vs. Ctr; ^#^ *P* < 0.05 vs. Cx43 WT G3w. One‐way ANOVA with Tukey's multiple comparisons test.

The degree of microglial activation in the optic nerve correlates with the severity of axonal degeneration.^[^ ^42^, ^43^ ^]^ In models of optic nerve injury, microglia facilitate axonal regeneration by phagocytosing myelin debris that obstructs axonal growth.^[^ ^44^ ^]^ Inhibition of microglial activation using minocycline ^[^ ^42^ ^]^ or anti‐TNF‐α antibodies ^[^ ^45^ ^]^ has been shown to prevent axonal degeneration and the death of RGCs. This evidence suggests that activated microglia may play a crucial role in RGC axonal degeneration associated with glaucoma. Our study demonstrated that Cx43 cKO in astrocytes influenced microglial activation, proliferation, migration, and phenotyping. We subsequently investigated the effects of these microglial alterations on optic nerve damage in the COH model. Neurofilament (NF) protein, a key component of the neuronal cytoskeleton, is predominantly found in neuronal cell bodies and axons, where it maintains normal cellular morphology.^[^ ^46^ ^]^ We employed NF antibodies to stain nerve fibers in vertical sections of the ONH within the COH model. **Figure** **5**a illustrates the schematic of the optic nerve section. Our results indicated that the nerve fiber thickness at the ONH‐retina junction was 62.8 ± 1.3 µm (n = 6) in the control group, exhibiting regular longitudinal alignment (indicated by red lines). In Cx43 WT mice at G1w, nerve fiber thickness was significantly reduced, displaying disorganized and discontinuous morphology. Conversely, in Cx43 cKO mice at G1w, nerve fiber thickness did not significantly differ from that of Cx43 WT mice. At G3w, nerve fiber thickness remained reduced in Cx43 WT mice, while Cx43 cKO mice exhibited a significant improvement in thickness (56.9 ± 3.6 µm, n = 6, *P* < 0.001), with restored regular longitudinal alignment (Figure 5b,c). These findings suggest that Cx43 cKO in astrocytes did not significantly influence nerve fiber damage at G1w in COH mice, but it significantly mitigated damage at G3w.

Changes in the axonal cytoskeleton can disrupt axonal transport. We further assessed the integrity of axonal transport by retrogradely labeling RGCs with cholera toxin subunit B (CTB), which was injected into the superior colliculus. As illustrated in Figure 5d–g, the control group exhibited 2,223.0 ± 77.0 CTB‐labeled RGCs per mm^2^ (n = 6). In the COH mice at G3w, the number of CTB‐labeled cells decreased to 1,190.0 ± 60.5 cells per mm^2^ (n = 6, *P* < 0.001). Conversely, in the Cx43 cKO mice, the number of CTB‐labeled cells increased to 1,697.0 ± 83.9 cells per mm^2^ (n = 6, *P* < 0.001). In the peripheral retina, the number of CTB‐labeled RGCs also decreased at G3w in COH mice compared to the control group; however, Cx43 cKO did not significantly affect these changes. These results suggest that conditional knockout of astrocyte Cx43 offers some protection for axonal transport in COH mice. To evaluate the survival of RGCs in Cx43 cKO mice, we performed Brn3a immunostaining in both central and peripheral retinal regions. At G3w, a significant reduction in the number of Brn3a‐positive RGCs was observed in the central and peripheral retinas of Cx43 WT mice compared to the control group. In Cx43 cKO mice at G3w, however, there was partial preservation of RGCs in both retinal regions. These findings suggest that Cx43 knockout in astrocytes provides partial protection against RGC loss in glaucomatous conditions (Figure 5h–j).

### 2.7 Construction of the hfCas13x Editing System and Efficient Knockdown of Cx43 in Retinal Macroglia

**Figure 6:**
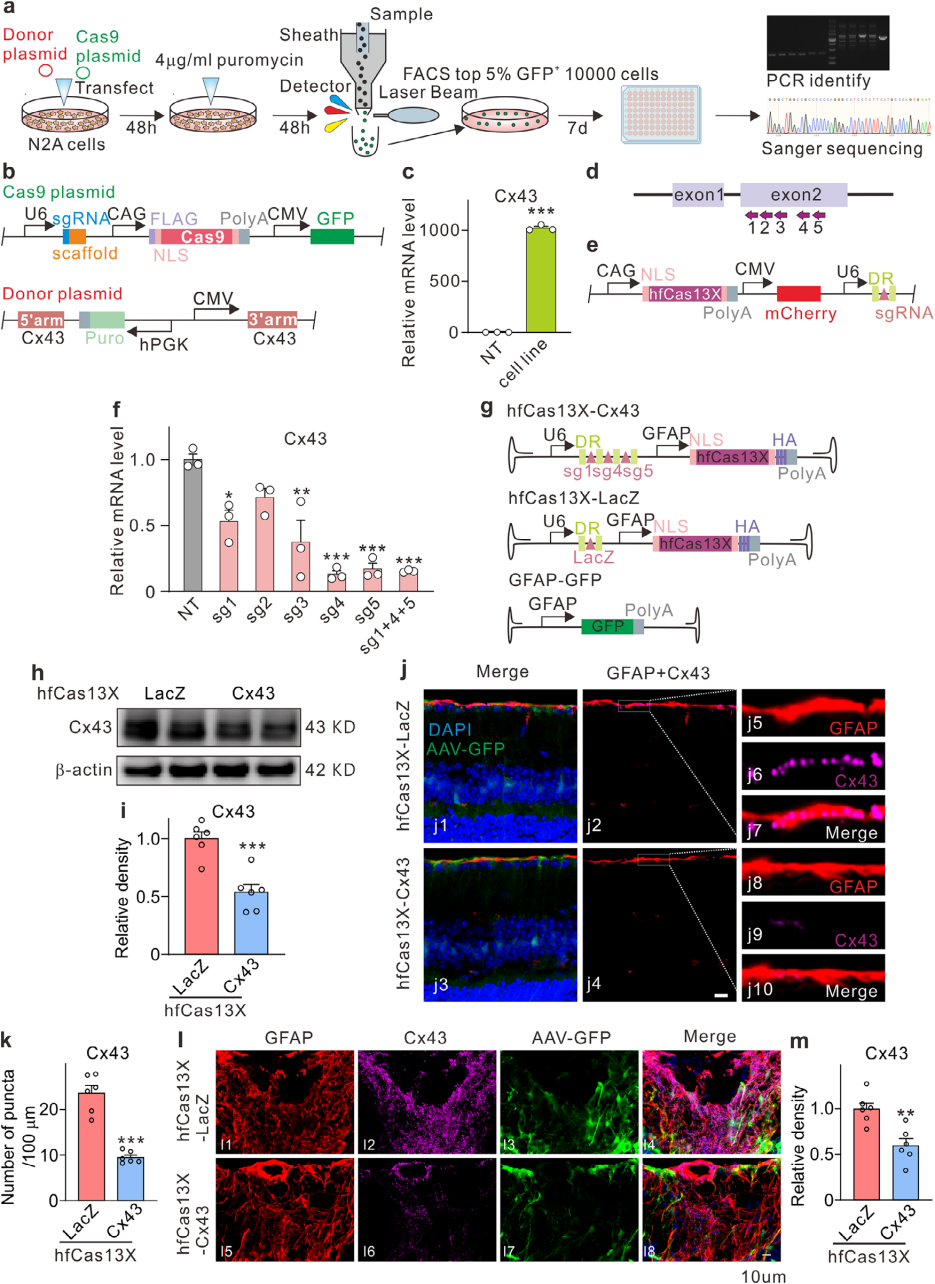
Development of the hfCas13x editing system and efficient Cx43 Knockdown in retinal macroglia. a) Schematic diagram of constructing stably transduced cell lines based on CRISPR Cas9 knock‐in system. b) Construct plasmids for stable transfection of N2a cell line, including sgRNA‐Cas9 expression vector and a vector containing donor fragment. c) Compared with the non‐treated (NT) group, the expression level of the Cx43 gene in the stably transfected N2a cell line was significantly increased. n = 3 biological replicates × 3 technical replicates. ^***^ *P* < 0.001 versus NT. Unpaired *t‐*test. d) The target locations of gRNA in Cx43 genes. e) Based on gRNA screening mediated by the hfCas13X editing system, hfCas13X and gRNA are expressed in the same plasmid vector. f) The efficiency of five different gRNAs in targeting and knocking down the Cx43 gene. n = 3 biological replicates × 3 technical replicates. ^*^ *P* < 0.05, ^**^ *P* < 0.01 and ^***^ *P* < 0.001 versus NT. Unpaired *t‐*test. g) The AAV virus vector encoding hfCas13X and gRNA. h,i) Representative images (h) showing the efficiency of CX43 knockdown by hfCas13X‐Cx43 in WT mice and quantification is shown in panel i. n = 6, ^***^ *P* < 0.001 versus hfCas13X‐LacZ group. Unpaired *t‐*test. j,k) Representative images (j) showing the efficiency of CX43 knockdown by hfCas13X‐Cx43 in the retina of WT mice, and quantification is shown in panel k. Scale bar: 10 µm. n = 6, ^***^ *P* < 0.001 versus hfCas13X‐LacZ group. Unpaired *t‐*test. l) Representative images showing the efficiency of CX43 knockdown by hfCas13X‐Cx43 in the ONH of WT mice. m) Bar chart showing the relative average intensity of Cx43 fluorescence signals in the ONH of mice. Scale bar: 10 µm for all images. n = 6, ^**^ *P* < 0.01 vs. hfCas13X‐LacZ group. Unpaired *t‐*test.

To achieve effective glaucoma treatment, we employed a novel RNA editing tool, the hfCas13x system, to knock down the Cx43 gene. Initially, we designed several 30‐nucleotide gRNA sequences targeting Cx43 using the Cas13 design platform available at https://cas13design.nygenome.org/. Given that Cx43 expression is low in N2a cells, we utilized the CRISPR/Cas9 technique to insert a CMV promoter and an hPGK‐puro‐polyA sequence upstream of the transcription start site (TSS) of the Cx43 gene in N2a cells via homologous recombination, thereby enhancing target gene expression (Figure S7, Supporting Information). Following transfection, successful knock‐in cells were selected using puromycin selection and flow cytometry, which resulted in a 1020‐fold increase in Cx43 mRNA levels (**Figure** **6**a–c). The established cell line was subsequently utilized to assess knockdown efficiency, with a co‐expression vector containing hfCas13x and gRNA transfected into the cells. To further improve knockdown efficiency, two Cx43 sgRNAs were tandemly linked, resulting in the hfCas13x system for Cx43 knockdown (Figure 6d–f).

We constructed the knockdown viral vector AAV‐GFAP‐hfCas13X‐Cx43 and the non‐targeting control virus AAV‐GFAP‐hfCas13X‐LacZ for subsequent studies (Figure 6g). These vectors were co‐injected subretinally with AAV‐GFP to label transduction efficiency. Three weeks post‐viral injection, we evaluated the safety of the viral vector. ERG results demonstrated no significant changes in the a and b‐wave between the hfCas13X‐Cx43 group, the LacZ group, and the WT mice (Figure S8, Supporting Information). Additionally, OCT and HE staining results indicated that viral injection did not affect the thickness or structure of the retina, as well as the structures of the heart, liver, spleen, lung, and kidney (Figure S9, Supporting Information). We also assess the knockdown effect via Western blot analysis. The results indicated that in normal WT mice, the injection of AAV‐GFAP‐hfCas13X‐Cx43 led to a 46.19% reduction in Cx43 expression levels compared to the LacZ virus group (Figure 6h,i). We used immunofluorescence to detect the expression of Cx43 in astrocytes in the retina and ONH, and the results showed that Cx43 was effectively knocked down (Figure 6j–m). Collectively, these experimental results suggest that subretinal injection of the hfCas13X‐Cx43 virus effectively induces RNA interference in macroglia, leading to targeted disruption of gene transcription without compromising the normal function of the retina, thereby ensuring systemic safety.

### 2.8 The hfCas13X‐Mediated Cx43 Knockdown Alleviates Damage to RGCs and Protects the Optic Nerve

**Figure 7:**
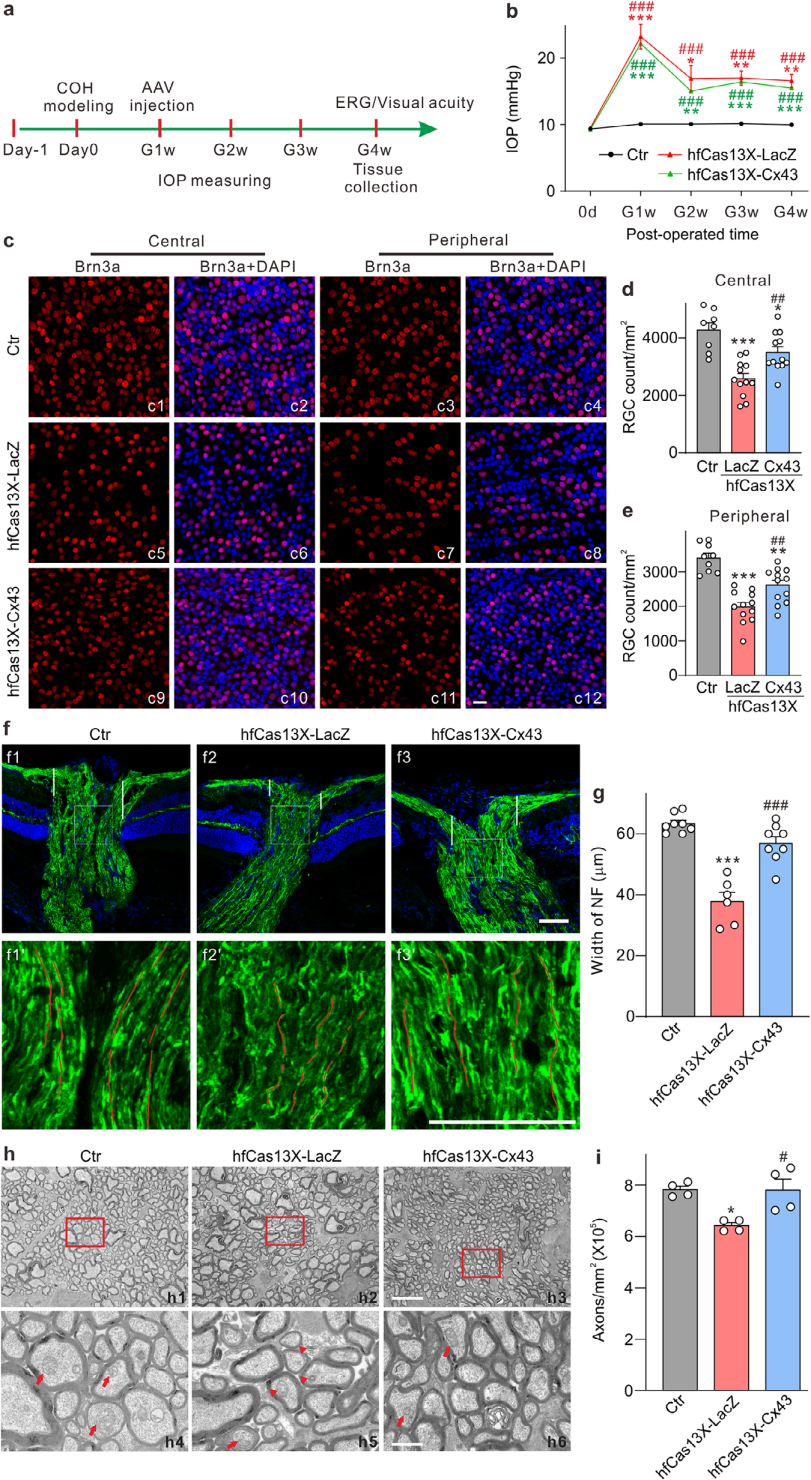
The hfCas13X‐mediated Cx43 knockdown in astrocytes increases RGC survival and reduces axonal degeneration in COH mice. a) The experimental strategy of magnetic beads‐induced COH mouse model. b) Changes in IOP in each group after magnetic beads modeling. ^*^ *P* < 0.05, ^**^ *P* < 0.01 and ^***^ *P* < 0.001 versus 0d. Non‐parametric Kruskal–Wallis ANOVA test. ^###^ *P* < 0.001 versus. unoperated eyes at the same time point. Unpaired *t*‐test with comparisons between each operated eye and unoperated eye. c) Representative micrographs of Brn3a‐positive RGCs from normal (Ctr) and COH flat‐mounted retinas at G4w with injections of hfCas13X‐LacZ and hfCas13X‐Cx43, respectively. Images were taken from the central and peripheral regions as shown in Figure 5d. Scale bar: 20 µm for all images. d,e) Quantification of Brn3a‐positive RGCs in eight fields under different conditions as shown in panel c. n = 8–12 retinas. ^*^ *P* < 0.05, ^**^ *P* < 0.01 and ^***^ *P* < 0.001 versus Ctr; ^##^ *P* < 0.01 versus hfCas13X‐LacZ. One‐way ANOVA with Tukey's multiple comparisons test. f) Representative micrographs showing the immunostaining of NF in the vertical slices of the ONH in Ctr, hfCas13X‐LacZ, and hfCas13X‐Cx43 group at G4w (f1–f3). f1’–f3’ are enlarged images of the white rectangular area in f1–f3. Scale bars: 50 µm. The red lines highlight the continuity of the neurofilament fibers. g) Bar charts showing the average width of the nerve fibers marked by the white line in panel f. n = 6–8 mice. ^*^ *P* < 0.05, ^**^ *P* < 0.01, ^***^ *P* < 0.001 versus Ctr; ^###^ *P* < 0.001 versus hfCas13X‐LacZ. One‐way ANOVA with Tukey's multiple comparisons test. (h, i) Representative TEM images h) and quantification i) of optic nerve cross‐sections of Ctr, hfCas13X‐LacZ, and hfCas13X‐Cx43 group at G4w. h4–h6 are enlarged images of the red rectangular area in f1–f3. Scale bar: 5 µm for h1–h3 and 1 µm for h4–h6. n = 4 mice. ^*^ *P* < 0.05 versus Ctr; *^#^P* < 0.05 vs. hfCas13X‐LacZ. One‐way ANOVA with Tukey's multiple comparisons test. The arrows point to intact mitochondria, which are characterized by a double‐membrane structure and cristae. The triangle markers indicate damaged mitochondria, which lack the cristae structure and show signs of mitochondrial degradation.

To investigate the therapeutic potential of Cx43 knockdown in glaucoma, we utilized the hfCas13X system to specifically target Cx43 in a glaucoma mouse model. The experimental group received the CRISPR‐hfCas13X virus targeting Cx43, while the control group was administered the LacZ virus. Treatment commenced 7 days following the induction of IOP elevation. Since the optimal efficacy period for AAV is achieved 3 weeks after the injections, functional assessments were conducted 3 weeks post‐treatment (G4w) (**Figure** **7**a). We measured the IOP weekly following the establishment of the model. After model establishment, the IOP in both the hfCas13X‐LacZ and hfCas13X‐Cx43 groups was significantly higher than that of the control group. However, there was no significant difference between the hfCas13X‐LacZ and hfCas13X‐Cx43 groups (Figure 7b). Survival of RGCs in both the central and peripheral regions of the retina (Figure 7c) at G4w was assessed through Brn3a immunostaining. The number of Brn3a‐positive RGCs was significantly diminished in both the central and peripheral retinal regions of the LacZ group compared to Ctr mice. This reduction is indicative of the typical RGC loss associated with elevated IOP in glaucoma. In contrast, treatment with the hfCas13X‐Cx43 virus significantly reduced RGCs’ death, suggesting that Cx43 knockdown provides neuroprotective benefits by preserving RGCs under glaucomatous conditions (Figure 7c–e). Further analysis demonstrated that the thickness of nerve fibers was substantially greater in the hfCas13X‐Cx43‐treated group compared to the LacZ group at 3 weeks post‐treatment (G4w) (Figure 7f,g). This preservation of nerve fiber integrity indicates that knocking down Cx43 aids in maintaining the structural health of axons, which are frequently compromised due to the degenerative effects of glaucoma. The ultrastructural changes in the optic nerve were further assessed using TEM, which revealed a significant increase in both the number and integrity of axons in the optic nerve of the hfCas13X‐Cx43‐treated group at G4w. High‐power images indicated that the hfCas13X‐LacZ group exhibited degraded mitochondria characterized by few ridges and a disrupted double‐layer membrane structure, whereas the hfCas13X‐Cx43 group displayed predominantly normal mitochondria (Figure 7h,i). Consequently, hfCas13X‐mediated astrocyte Cx43 knockdown effectively mitigated RGC loss and axonal degeneration in the retinas of mice with chronic ocular hypertension.

**Figure 8:**
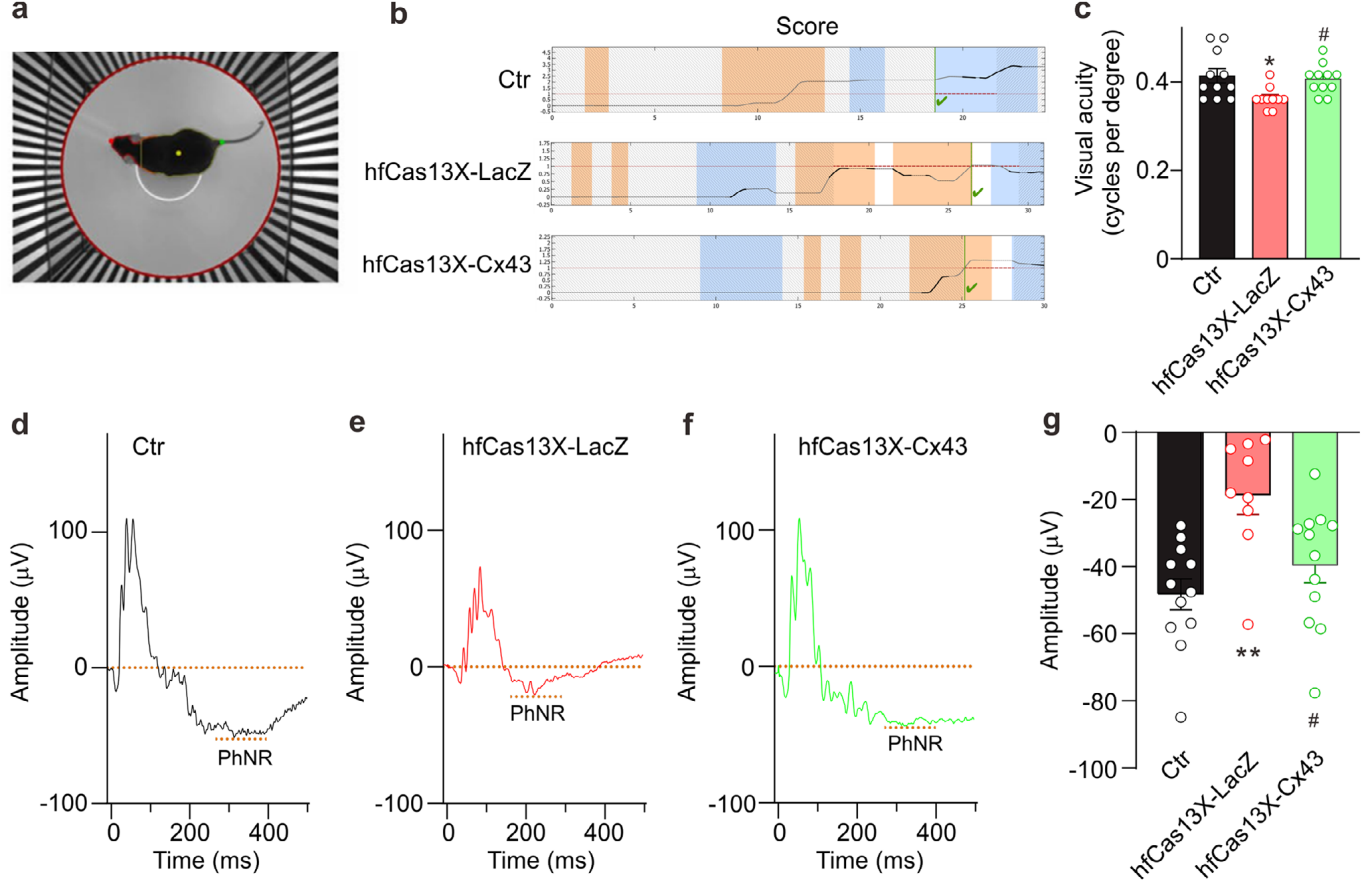
Effects of hfCas13X‐mediated astrocyte Cx43 knockdown on visual function in COH mice. a) The mouse is located in a virtual rotating drum center with a black and white stripe. The drum rotates clockwise or counterclockwise with 12 /s of velocity. b) The head movement score was assessed under various conditions. A successful trial of the optomotor response during the experimental trials is defined as one in which the head movement score surpasses the chance level of stimulus‐independent head motion. The *x*‐axis represents the time of the trial (in seconds), and the *y*‐axis represents the head movement score. The colored regions represent different phases of the optomotor response. The orange region corresponds to the phase where the stripes are rotating clockwise (rightward), while the blue region corresponds to the phase where the stripes are rotating counterclockwise (leftward). The green checkmarks indicate successful trials where the head movement score surpasses the threshold for stimulus‐independent head motion, indicating a positive optomotor response. The graph shows the head movement responses over time for each experimental group, reflecting varying levels of visual acuity between the groups. c) Quantification of visual acuity measuring the opto‐motor reflex expressed as cycles per degree of Ctr, hfCas13X‐LacZ, and hfCas13X‐Cx43 mice at G4w. n = 10 ‐11 mice. ^*^ *P* < 0.05 versus Ctr; ^#^ *P* < 0.05 versus hfCas13X‐LacZ. Non‐parametric Kruskal–Wallis ANOVA test. d–f) Typical ERG traces were recorded from Ctr (d), hfCas13X‐LacZ (e), and hfCas13X‐Cx43 (f) mice at G4w. g) Bar charts showing the PhNR amplitudes under different conditions. n = 9–12 mice. ^**^ *P* < 0.01 versus Ctr; ^#^ *P* < 0.05 versus hfCas13X‐LacZ. One‐way ANOVA with Tukey's multiple comparisons test.

To determine whether hfCas13X‐mediated astrocyte Cx43 knockdown is sufficient to restore impaired visual function, we compared the optomotor reflex response (OMR) of the animals. This was assessed by tracking the unconditional compensatory head movements of the animals as they attempted to stabilize images of their moving environment, represented by rotating vertical stripes.^[^ ^47^, ^48^ ^]^ The visual acuity of mice in the hfCas13X‐LacZ group significantly declined by G4w, while those in the hfCas13X‐Cx43 group were maintained at the control levels (**Figure** **8**a–c). Finally, we employed ERG experiments to assess whether the knockdown of astrocyte Cx43 mediated by hfCas13X could enhance visual function in COH mice. The PhNR wave serves as an indicator of the functional status of RGCs.^[^ ^49^, ^50^ ^]^ As illustrated in Figure 8d–g, at G4w, subretinal injection of the hfCas13X‐LacZ virus resulted in a significant reduction in the PhNR amplitude of COH mice compared to the control group. Furthermore, the hfCas13X‐LacZ group exhibited a partial recovery of PhNR amplitude when compared to the hfCas13X‐LacZ group, and a significant difference was also noted when compared to the control group. This enhancement in visual function underscores the potential of Cx43 knockdown to translate structural preservation into meaningful functional benefits, thereby providing a promising strategy for mitigating glaucomatous damage.

## 3 Discussion

This study presents compelling evidence that Cx43 is integral to the pathogenesis of glaucoma, primarily by modulating microglial activation via macroglial cells. Our findings suggest that Cx43 plays a significant role in the neuroinflammatory processes that lead to the degeneration of RGCs in glaucoma. Utilizing the advanced hfCas13X RNA editing system, we demonstrate that the targeted downregulation of Cx43 can effectively alleviate these pathogenic processes, thereby providing a novel therapeutic strategy to slow the progression of glaucoma.

Our research utilizing the COH experimental glaucoma mouse model revealed that as IOP increases, astrocytes display activated morphological characteristics by G2d. Concurrently, the number of microglia in the NFL and the ONH increases by G2d, with observable activation by G3d. These results suggest that astrocyte activation may precede microglial activation, thereby inducing microglial proliferation and/or migration to the NFL and the ONH. The observed increase in microglial numbers within the NFL may be attributed to a combination of proliferation and migration. Our previous studies have demonstrated that in the glaucomatous retina, ATP released by Müller cells through Cx43 activates microglia via the P2×7R, leading to their activation, proliferation, and/or migration, with migration primarily regulated by the P2×4R.^[^ ^3^, ^4^ ^]^ We have confirmed a similar mechanism in astrocytes, where ATP released through Cx43 channels is crucial for microglial activation, proliferation, and/or migration.

It is noted that astrocytes can shift from A2 to A1 phenotype in response to injury or diseases.^[^ ^2^ ^]^ In this study, we did not pay attention to the phenotype shift of astrocytes. Based on our results, astrocytes were activated in COH mice, and Cx43 intervention in astrocytes attenuated microglial activation. We speculate that A1‐type astrocytes may mediate these effects. Certainly, future studies should be conducted to address this issue.

Activated microglia can differentiate into pro‐inflammatory M1‐like or anti‐inflammatory M2‐like phenotypes, and this phenotypic conversion is crucial in the pathogenesis of neurodegenerative diseases. During the early stages of IOP elevation, there is a temporary increase in the number of CD206‐marked M2‐like microglia. However, over time, the number of CD86‐marked M1‐like microglia surpasses that of M2‐like microglia, resulting in M1‐like microglia becoming the dominant phenotype. The Cx43 cKO in astrocytes has been shown to regulate this microglial phenotype conversion by increasing the proportion of M2‐like microglia while decreasing the proportion of M1‐like microglia. The P2×7R is associated with the pro‐inflammatory phenotype of microglia, whereas the P2Y12R is linked to the anti‐inflammatory phenotype.^[^ ^27^, ^28^ ^]^ In our study, we observed that the Cx43 cKO in astrocytes led to a reduction in P2×7R expression and an increase in P2Y12R expression. The decreased stimulation of the P2×7R may attenuate the activity of the NF‐κB signaling pathway, thereby reducing the proportion of pro‐inflammatory M1‐like microglia. Simultaneously, elevated ATP levels may activate the P2Y12R and its downstream STAT6 pathway, facilitating the conversion of microglia to the anti‐inflammatory M2‐like phenotype.^[^ ^26^ ^]^ M2‐like microglia play a significant role in neuroprotection by phagocytosing cellular debris, promoting extracellular matrix reconstruction, and supporting neuronal survival and regeneration, while a reduction in M1‐like microglia alleviates neuroinflammation. Collectively, these mechanisms contribute to the neuroprotective effects observed with Cx43 knockout in astrocytes, indicating that Cx43‐mediated ATP release by astrocytes is a key regulator of microglial activation and migration in glaucoma.

Astrocytes are the primary glial cells in the central nervous system (CNS). Changes in astrocyte morphology and function are closely associated with neurodegenerative diseases such as Alzheimer's disease, depression, Parkinson's disease, and glaucoma. Intervening in disease‐related genes and their signaling pathways in astrocytes may provide new approaches for treating these diseases.^[^ ^51^, ^52^ ^]^ Our study reveals that Cx43 cKO in astrocytes provides significant protection in late‐stage COH mice, as evidenced by increasing the proportion of M2‐like microglia, decreasing the proportion of M1‐like microglia, and enhancing nerve fiber integrity. Our gene therapy was also administered via subretinal injection 1 week after establishing the high IOP model. As anticipated, hfCas13X‐mediated Cx43 knockdown in macroglial cells significantly increased RGC survival and nerve fiber integrity, with notable improvements in visual function.

During the pathogenesis of glaucoma, the expression of Cx43 undergoes dynamic changes that have distinct impacts on neuronal health at different stages of the disease. In the early stages of glaucoma, Cx43 expression may confer protective effects through multiple mechanisms. For example, the Cx43 network can provide neuroprotection during metabolic stress by redistributing resources, regulating extracellular concentrations of glutamate and K^+^, and preventing hyperexcitable or damaged neurons from inducing abnormal excitability in neighboring cells.^[^ ^12^, ^53^ ^]^ However, as glaucoma progresses, particularly in the later stages, the role of Cx43 may shift toward promoting neuronal damage. Research indicates that increased formation of gap junctions by Cx43 in models of retinal ischemia/reperfusion injury can facilitate the spread of inflammatory molecules between cells, thereby exacerbating damage to RGCs.^[^ ^14^ ^]^ Furthermore, substantial ATP released by astrocytes via Cx43 can stimulate microglia to adopt a pro‐inflammatory M1‐like phenotype, which accelerates neuroinflammation and RGC apoptosis. Consequently, in late‐stage glaucoma, the expression and function of Cx43 may transition from neuroprotection to neurodegeneration. These studies suggest that targeting the regulation of Cx43 is emerging as a promising therapeutic strategy for glaucoma, particularly in its later stages, where mitigating ATP release from macroglial cells and the associated dissemination of pro‐inflammatory mediators could offer significant neuroprotection.

In various neurodegenerative diseases, targeting Cx43 has demonstrated favorable outcomes in both preclinical and clinical studies. For instance, in models of ischemic brain injury, inhibitors of Cx43 gap junction proteins have been effective in reducing brain inflammation and neuronal damage.^[^ ^54^ ^]^ Furthermore, the application of inhibitors to downregulate Cx43 expression in experimental models of stroke and epilepsy has been shown to diminish neuronal damage and inflammation in the affected regions.^[^ ^55^ ^]^ Xiflam, an oral connexin hemichannel blocker, has shown evidence of safety in more than 1,000 patients and is in preparation for phase 2 clinical trials. Aims to ameliorate the progression of many devastating chronic inflammatory diseases of the eye and other parts of the body by modulating connexin hemichannels.^[^ ^56^ ^]^ These investigations underscore the significance of Cx43 as a vital therapeutic target across a spectrum of diseases and provide essential references for its potential application in glaucoma therapy.

Currently, gene therapy for glaucoma primarily targets the reduction of IOP through the trabecular meshwork or aqueous outflow pathways. For instance, therapies based on CRISPR‐Cas9 have been developed to knock out genes associated with elevated IOP, including *MYOC*, *AQP1*, and *CA2*.^[^ ^57^, ^58^, ^59^ ^]^ In contrast to the Cas9 system, the CRISPR‐CasRx system targets RNA rather than DNA, allowing for the regulation of gene expression without modifying the genome. In the context of glaucoma therapy, the CasRx system predominantly focuses on the RNA‐level knockdown of aquaporin 1 (Aqp1), β2‐adrenergic receptor (Adrb2), and Rho‐associated kinase 1/2 (Rock1/Rock2). By managing IOP through this mechanism, the CasRx system effectively reduces aqueous humor production and modulates the signaling pathways linked to elevated IOP.^[^ ^60^ ^]^ These therapies aim to mitigate mechanical stress‐induced damage to the optic nerve by enhancing aqueous outflow and lowering IOP. However, while promising, they do not directly address the neurodegenerative processes that continue to harm RGCs even after IOP reduction. Our study represents the first application of the advanced hfCas13X RNA editing system to specifically target Cx43 in macroglial cells, achieving significant neuroprotective effects in a glaucoma mouse model. The hfCas13X system is a novel CRISPR‐Cas13 variant that offers a high‐fidelity, RNA‐specific editing method, thereby avoiding permanent genomic alterations and minimizing potential off‐target effects. Although the CasRx system has shown efficacy in RNA editing, its application in vivo may pose greater risks of side effects, including unintended degradation of non‐target RNAs, which could lead to cytotoxicity and other adverse reactions. In the realm of neuroprotection, the hfCas13X system, due to its high fidelity and reduced side effects, exhibits greater potential.^[^ ^17^, ^18^ ^]^ This advantage is particularly important in the treatment of chronic neurodegenerative diseases such as glaucoma, where long‐term targeted therapy necessitates the minimization of non‐specific effects.

In comparison to traditional therapies, this innovative approach presents several advantages, including the potential for long‐lasting effects from a single treatment and the mitigation of patient compliance challenges. Furthermore, RNA editing tools such as hfCas13X are deemed safer and more reversible than DNA‐targeting CRISPR systems. The documented reduction in RGC death and the preservation of optic nerve axon integrity further underscore the therapeutic potential of this method. While we provide compelling evidence for the neuroprotective effects of Cx43 knockdown in the preservation of visual function, additional studies are required to optimize this strategy for clinical application. Future investigations should prioritize the assessment of the long‐term safety and efficacy of hfCas13X‐mediated Cx43 knockdown in larger animal models and non‐human primates. Moreover, exploring the integration of this gene therapy with existing IOP‐lowering treatments could yield a comprehensive strategy for glaucoma management. A thorough understanding of the precise mechanisms by which Cx43 influences glial cell activation, along with the identification of the optimal timing for intervention, will be critical for the successful translation of this therapy to human patients.

In conclusion, our study demonstrates that hfCas13X‐mediated Cx43 knockdown in macroglia represents a novel and effective strategy for treating glaucoma. This approach reduces neuroinflammation and preserves RGC integrity, indicating significant potential for mitigating the neurodegenerative effects of glaucoma and preserving vision. These findings pave the way for further exploration of RNA‐based gene therapies in the treatment of neurodegenerative diseases.

## 4 Experimental Section

### Animals

The handling of experimental animals in this study adhered to the Guide for the Care and Use of Laboratory Animals as established by the National Institutes of Health (NIH) and received approval from the Laboratory Animal Ethics Committee of Fudan University (approval No: 20220228‐158). Efforts were made to minimize pain and discomfort for the experimental animals throughout the duration of the experiment. Male C57BL6 mice, all over six weeks old, were procured from the SLAC Laboratory Animal Company (Shanghai, China). Additionally, hGFAP‐cre and Cx43^flox/flox^ mice were generously provided by Professor Lan Xiao from Army Medical University (formerly known as the Third Military Medical University). The P2xr7^tm1Gab^ transgenic mice (*P2×7R^−/−^* mice) were kindly donated by Professor Yu‐Qiu Zhang from the Institute of Brain Science at Fudan University. Both sexes of the transgenic mice were used in this study. The experimental mice were housed in a controlled environment featuring a 12‐h light/dark cycle, with the temperature maintained at 21°C, ensuring adequate access to food and water.

### COH Model in Mice

A microbead occlusion model was employed to induce COH, as described in the previous study.^[^ ^10^ ^]^ Briefly, 2 µL of micromagnetic beads (Cat#BM547, Bangs Laboratories, Indianapolis, IN, USA), ≈9 µm in diameter, were injected into the anterior chamber of the anesthetized mouse (CAS#57‐33‐0, 0.6% pentobarbital sodium (Merck, Kenilworth, NJ, USA), 10 µL g^−1^, intraperitoneal injection) using an ophthalmic surgical microscope (Carl Zeiss, Jena, Germany). An equal volume of NS was administered as a control. IOP measurements were standardized to occur between 9 and 10 a.m. to mitigate the potential effects of circadian rhythm variations on IOP. IOP of mice under anesthesia was assessed using a handheld Tonolab rebound tonometer (Icare, Helsinki, Finland). The average of five consecutive measurements with a deviation within 5% was recorded. IOP was measured preoperatively and on days 0 (G0d), 2 (G2d), 3 (G3d), 4 (G4d), and weekly (up to 4 weeks, G1w to G4w) post‐injection.

### Intravitreal Injections

The procedures for intravitreal injection were basically similar to those in the previous reports.^[^ ^3^, ^10^ ^]^ In short, a small incision ≈1–2 mm below the corneoscleral limbus of the anesthetized mouse was created using the needle of a 1 mL syringe. Subsequently, a microsyringe (Hamilton, Reno, NV, USA) was employed to insert the needle obliquely downward at a 45° angle into the vitreous cavity, near the ONH. 2 µL of the drug were slowly injected into the vitreous cavity. Depending on the specific experimental requirements, different drugs may be injected. The concentrations of the injected drugs were as follows: Gap26 (Cat#A1044, 200 µm, ApexBio, Houston, USA), ARL67156 (Cat#A265, 100 µm, Sigma–Aldrich, St. Louis, MO, USA), LAA (Cat#A7275, 6 mm, Sigma–Aldrich),^[^ ^61^ ^]^ and 5‐BDBD (Cat#SML0450, 10 µm, Sigma–Aldrich).^[^ ^4^, ^10^ ^]^ The control group will receive the same volume of NS. The intravitreal injection of drugs should occur one day prior to the COH model operation to ensure adequate healing of the wound for the successful establishment of the model.

### Subretinal Injections

A small scleral incision was made at the limbus using a 30‐gauge needle in the anesthetized mouse under the aid of an ophthalmic surgical microscope (Carl Zeiss). Subsequently, 1.5 µL of either AAV‐GFAP‐hfCas13X‐Cx43 or AAV‐GFAP‐hfCas13X‐LacZ was injected into the subretinal space with a 33‐gauge Hamilton syringe. The successful delivery was confirmed by the formation of a retinal bleb.

### Immunofluorescence Staining

Immunofluorescence staining was performed following the procedures described previously.^[^ ^10^, ^62^ ^]^ Briefly, the mouse optic cup was fixed in 4% paraformaldehyde at 4°C for 4 h. Following fixation, the tissue was dehydrated in sucrose and embedded in OCT compounds, and retinal vertical sections, with a thickness of 14 µm, were obtained using a frozen microtome (Leica, Nussloch, Germany). The primary antibody was incubated with a solution containing 0.1% Triton X‐100, 5% normal donkey serum, and 1% BSA at room temperature for 1.5 h, followed by an overnight incubation for one day. The primary antibodies utilized included rabbit anti‐Cx43 (Cat#ab11370, 1:1000, Abcam, Cambridge, MA, USA), mouse anti‐GFAP (Cat#G6171, 1:400, Sigma–Aldrich, USA), rabbit anti‐GFAP (Cat#Z0334, 1:1000, DAKO, Denmark), rabbit anti‐IBA1 (Cat#ab178846, 1:500, Abcam, Japan), goat anti‐IBA1 (Cat#27991,1:500, Wako), rabbit anti‐TSPO (Cat#ab109497, 1:1000, Abcam), mouse anti‐CRE (Cat#MAB3120, 1:1000, Sigma), rabbit anti‐CD206 (Cat#ab64693, 1:500, Abcam), rat anti‐CD86 (Cat#553689, 1:100, BD Biosciences, USA), chicken anti‐NF (Cat#ab4680, 1:3000, Abcam), rabbit anti‐GS, (Cat#ab49873, 1:1000, Abcam) and rabbit anti‐Brn3a (Cat#ab245230, 1:500, Abcam). After washing with phosphate buffer saline, the sections were incubated with Alexa fluorescently coupled secondary antibody Cy3‐conjugated IgG Donkey anti‐rabbit/ Mouse (Cat#711‐165‐152/715‐165‐150, 1:400, Jackson ImmunoResearch Laboratories, West Grove, USA), Alexa Fluor 488 ‐conjugated IgG Donkey Anti‐Rabbit/Mouse/Goat (Cat#711‐545‐152/715‐545‐150/705‐545‐003, 1:400, Jackson ImmunoResearch Laboratories), Cy5‐conjugated IgG Donkey Anti‐Rabbit (Cat#711‐175‐152, 1:400, Jackson ImmunoResearch Laboratories), Alexa Fluor® 488 goat anti‐chicken (Cat#2180688, 1:400, Invitrogen, CA, USA), Cy3‐conjugated IgG Goat anti‐rat (Cat#A0507, 1:400, Beyotime, Shanghai, China), at room temperature for 1.5 h, protected from light. 4',6‐diamidino‐2‐phenylindole (DAPI) (Cat#28718‐90‐3, Roche, Basel, Switzerland) was used for nuclear staining. Images were captured at 1.0 µm intervals using the FV1000 confocal laser scanning microscope (Olympus, Monolith, Tokyo, Japan). The mean fluorescence intensity of each micrograph was quantified by using ImageJ software (version 1.8.0, NIH, Bethesda, USA). The data were presented as the integrated optical density (IOD)/area. For vertical sections of the ONH, 2–3 images per animal were analyzed. For the whole flat‐mounted retina, eight fields (Figure 5d) were selected from the center (≈0.8 mm from the ONH), and the peripheral (≈1.6 mm from the ONH), and the fluorescence intensity or the number of Brn3a‐positive cells were analyzed and counted. For retinal vertical sections, 1–2 sections per animal were chosen, and the numbers of Cx43 puncta in 4–6 randomly selected fields in each section were counted and averaged.

### Western Blot Analysis

Western blotting was performed using the procedures described previously.^[^ ^10^, ^63^, ^64^ ^]^ In short, retinal tissue was cleaved in a RIPA buffer containing protease and phosphatase inhibitors. Following homogenization and centrifugation, the protein concentration was determined using the BCA protein assay kit (Cat#A55865, Thermos Fisher Scientific, Waltham, MA, USA). Protein samples (10 µg) were loaded onto a 10% SDS‐PAGE gel and subsequently transferred to PVDF membranes. The membranes were sealed with 5% skim milk for 2 h. They were then incubated overnight with primary antibodies rabbit anti‐Cx43 (Cat#ab11730, 1:1000, Abcam), rabbit anti‐P2×4R (Cat#APR‐024, 1:1000, Alomone Labs, Israel), rabbit anti‐P2×7R (Cat#APR‐012, 1:1000, Alomone Labs), rabbit anti‐P2Y12R (Cat#APR‐012, 1:1000, Alomone Labs), mouse anti‐GFAP (Cat#G6171, 1:400, Sigma–Aldrich), rabbit anti‐TSPO (Cat#ab109497, 1:1000, Abcam) and mouse anti‐β‐actin (Cat#A5441,1:500, Sigma–Aldrich). Following washes, HRP‐conjugated secondary antibodies: donkey anti‐mouse/anti‐rabbit (Cat#715‐035‐150/711‐035‐152, 1:5000, Jackson ImmunoResearch Labs) were applied for 1.5 h at room temperature. Detection was performed using ECL, and quantification was carried out with ImageLab.

### Retrograde Labeling of RGCs with CTB

To assess the integrity of axoplasmic transport, Alexa Fluor 594‐conjugated CTB (Cat#C34777, Thermo‐Fisher Scientific, USA) was injected into the superior colliculus to retrogradely label RGCs.^[^ ^62^ ^]^ The anesthetized mouse was immobilized on a stereotaxic apparatus from the Bregma point; measurements of 3.9 mm backward, 0.8 mm sideways, and 1.5 mm downward were taken to locate the superior colliculus. A CTB solution containing 500 nL of 1% Alexa Fluor 594 was then injected into the superior colliculus using a microinjector (Drummond Scientific Company, USA). Five days post‐operation, the retina was harvested, fixed in 4% paraformaldehyde, and subsequently scanned under a fluorescence microscope (Nikon, Corporation, Tokyo, Japan) across the entire field. The red fluorescence signals in eight fields randomly captured in the central and peripheral regions of each retina were quantified.^[^ ^10^ ^]^ Imaging was performed using FV1000 confocal laser scanning microscopy (Olympus, Japan). Dyeing and counting were conducted by separate personnel.

### Plasmid Construction

An all‐in‐one plasmid vector containing hfCas13X and U6‐gRNA was utilized.^[^ ^17^ ^]^ The gRNAs were synthesized as single‐stranded DNA oligonucleotides (refer to Table S1, Supporting Information for all gRNA sequences). The gRNA oligonucleotide was annealed under the U6 promoter using the Bpil enzyme (Cat#FD1014, Thermos Fisher Scientific) to construct the expression vector for the hfCas13X system. The mouse Cx43 genome sequence (NC_000076.7) was obtained from the US National Center for Biotechnology Information. The Cas9 sgRNA sequence was designed to be located 50 base pairs upstream of the transcription start site of the target gene. Selected sgRNAs were synthesized as oligonucleotides and cloned into the HP180‐U6‐sgRNA‐CMV‐Cas9‐CMV‐EGFP plasmid. For the assembly of the donor plasmid, an 800 bp sequence on both sides of the Cas9 nick site was used as the homology arm, into which the hPGK‐PuroR‐BGH polyA fragment and the CMV promoter sequence were inserted, followed by assembly of the aforementioned fragments through Gibson assembly.

### Production of Adeno‐Associated Virus (AAV)

All AAVs utilized in this study were packaged by OBiO Technology (Shanghai, China). The hfCas13X and sgRNA sequences were integrated into the AAV vector, with hfCas13X being driven by the GFAP promoter. The knockdown viral vector AAV8‐GFAP‐hfCas13X‐Cx43 and the non‐targeting control virus AAV8‐GFAP‐hfCas13X‐LacZ were constructed. The virus titer was measured at 2E+13 GC/ml.

### N2a Cell Culture and Transfection

The N2a cell line was purchased from the cell bank of the Shanghai Institute of Biochemistry and Cell Biology, Chinese Academy of Sciences. N2a cells were cultured in DMEM (Cat#11965092, Gibco, Life Technologies, Rockville, MD, USA) supplemented with 10% fetal bovine serum (Cat#A5670701, Gibco) and 1% penicillin‐streptomycin (Cat#10378016, Gibco). The cells were maintained at 37°C with 5% CO_2_. N2a cells were transfected with plasmids using PEI. After 72 h of transfection, the top ≈30% of 50,000 mCherry^+^ cells were sorted using a BD FACS Aria III flow cytometer (BD Biosciences, San Jose, CA, USA) and lysed for quantitative PCR (qPCR) analysis.

### Stable Cell Line Culture

The Cx43‐targeting vector HP180‐U6‐sgRNA‐CMV‐Cas9‐CMV‐EGFP was co‐transfected with the vector expressing the donor fragment into N2a cells using Lipofectamine 3000 (Cat#L3000001, Thermos Fisher Scientific), following the manufacturer's instructions. Two days post‐transfection, puromycin (Cat#60209ES10, YEASEN, Shanghai, China) was added to the culture medium, achieving a final concentration of 4 µg mL^−1^. Four days after the transient transfection, the cells were digested using 0.05% trypsin, and 10,000 mixed clone cells were sorted with a BD FACS Aria III flow cytometer and subsequently placed into a 10 cm culture dish for continued growth, maintaining the medium with a concentration of 4 µg mL^−1^ of puromycin. When a single cell divided and developed into a cluster of several cells—≈1 week later—a single clone was selected and transferred to a 96‐well plate for further culture, at which point puromycin treatment was discontinued. Upon reaching the appropriate passage level, 20 well samples were selected for PCR identification and sequencing.

### qPCR

Total RNA was extracted from cultured cells using TRIzol reagent (Cat#15596018, Invitrogen, CA, USA).^[^ ^10^ ^]^ cDNA was synthesized by HiScript Q RT SuperMix for qPCR (Cat#R42301, Vazyme, Nanjing, China). qPCR was performed using AceQ Universal SYBR qPCR Master Mix (Cat#Q11102, Vazyme) on a Roche 480 II‐A (Roche, Basel, Switzerland). The primers used in this study were Gja1‐F (CTGAGTGCGGTCTACACCTG), Gja1‐R (GAGCGAGAGACACCAAGGAC), actin‐F (GGCTGTATTCCCCTCCATCG), actin‐R (GGCTGTATTCCCCTCCATCG). Relative expression levels of target genes were calculated using the 2^^−ΔΔCt^ method, with β‐actin as the internal control.

### RNA Sequencing (RNA‐seq)

Retinal RNAs from WT, Cx43 WT G3w, and CX43 cKO G3w mice were extracted for RNA sequencing and initial bioinformatics analysis by Genewiz (Azenta Life Sciences, Chelmsford, MA, USA). The mRNA was fragmented using divalent cations and high temperature, followed by priming with random primers. First‐strand and second‐strand cDNA synthesis was performed. The purified double‐stranded cDNA was then subjected to end repair and the addition of ‘A’ tails in a single reaction, followed by TA ligation to attach adapters to both ends. Size selection of the adapter‐ligated DNA was conducted using DNA Clean Beads (Cat#N41101, Vazyme). Each sample underwent PCR amplification with P5 and P7 primers, and the PCR products were subsequently verified. Different indexed libraries were multiplexed and loaded onto an Illumina Novaseq 6000 instrument (Illumina, Inc., San Diego, CA, USA), where sequencing was carried out using a 2 × 150 paired‐end (PE) configuration in accordance with the manufacturer's instructions. Differential expression analysis was conducted using the DESeq2 Bioconductor package, which relies on a model based on the negative binomial distribution. The dispersion estimates and log‐fold changes were informed by data‐driven prior distributions. A Padj threshold of ≤ 0.05 was applied to identify significantly differentially expressed genes (DEGs). GO enrichment analysis of the DEGs was performed using R software version 3.2.0 (R Foundation for Statistical Computing, Vienna, Austria), employing hypergeometric distribution to identify significantly enriched terms. Additionally, GSEA was carried out using GSEA software from the Broad Institute (MA, USA).

### Electroretinography (ERG)

ERG was recorded following an overnight dark adaptation of the mice, with pupil dilation achieved using 0.5% tropicamide eye drops. The mice were under deep anesthesia, administered with sodium pentobarbital at a dosage of 70 mg kg^−1^. Dark‐flash ERG recordings were obtained during the dark adaptation phase, utilizing a range of white flashes with intensities of 3 CD s m^−^^2^. Light‐flash ERG recordings were obtained during the light adaptation phase, utilizing a range of white flashes with intensities of 10 CD s m^−^^2^. The analysis concentrated on evaluating the amplitudes of a and b‐waves. Subsequently, the recording protocol incorporated a brief red stimulus of 20 CD s m^−^^2^ superimposed on a white light background of 30 CD s m^−^^2^. The flash duration was set to 5 ms, with a frequency of 0.5 Hz. This procedure resulted in the recording of a response known as the photonegative response (PhNR), defined as the amplitude difference between the baseline level prior to stimulation and the trough following the b‐wave.^[^ ^49^ ^]^

### Spectral‐Domain Optical Coherence Tomography (SD‐OCT)

After anesthesia and pupil dilation, mice were correctly positioned, and OCT scans were performed. Images were automatically captured using an optical coherence tomography scanner (F5100, Optoprobe, Fremont, CA, USA). ImageJ software was used to measure and analyze retinal thickness.

### Optokinetic Response Test

To evaluate the visual acuity of freely moving mice, the OptoDrum system (Striatech GmbH Vor dem Kreuzberg, Tübingen, Germany) was employed to assess photodynamic reflection.^[^ ^65^ ^]^ In this setup, mice were positioned on an elevated platform surrounded by monitors, where they were exposed to a stripe pattern characterized by maximum contrast and a constant rotation speed of 12° s^−1^. The OptoDrum software (version 1.2.6) facilitated unbiased automatic detection and analysis of the behavior of mice, continuously adjusting the stimulation patterns (cycles) to determine the visual acuity threshold of the animals.

### HE Staining

The eyeballs were fixed in paraffin‐embedded FAS eyeball fixative and sectioned to a thickness of 6 µm. Hematoxylin and eosin (H&E) staining of the retinal sections was performed using a specialized staining kit (Sigma–Aldrich). Images for histopathological analysis were acquired using an Olympus VS120 microscope.

### Transmission Electron Microscope (TEM)

The optic nerve was dissected and fixed using 2.5% glutaraldehyde (G1102, Servicebio, Shanghai, China). Subsequently, 80 nm ultrathin sections of the optic nerve segments were collected and examined using a transmission electron microscope (TEM, HT7800, Hitachi, Japan).^[^ ^66^ ^]^ Quantitative calculations and analyses of axonal evaluations were conducted.

### Statistical Analysis

Each experiment included at least three animals per group, typically with a minimum of five animals (n ≥ 5). For Western blot analysis, all data were normalized to their corresponding β‐actin and then to the control. For immunofluorescence analysis, all data on immunofluorescence intensity were normalized to control. All data were presented as mean ± SEM. Data analysis was conducted using GraphPad Prism software (version 10.0; GraphPad Software, Inc., San Diego, CA, USA). The Shapiro–Wilk test was employed to assess data distribution. For two independent experimental groups, a two‐tailed unpaired *t*‐test was employed when the normal distribution was satisfied. In contrast, the non‐parametric Mann–Whitney test was utilized when the normal distribution was not satisfied. For three or more independent experimental groups, One‐Way ANOVA was applied when the normal distribution was met, followed by Tukey–Kramer multiple comparison analysis for post hoc testing. Conversely, the non‐parametric Kruskal–Wallis test was used when the normal distribution was not satisfied, with Dunnett's multiple comparison analysis serving as the post–hoc test. A *p‐*value of less than 0.05 was considered statistically significant.

## Conflict of Interest

The authors declare no conflict of interest.

## Author Contributions

G.Z. and Z.L. contributed equally to this work. Z.W. and G.Z. jointly conceptualized the study and designed the experiments. G.Z., Z.L., S.Y.L., Q.X., Y.W., Y.L.L., Y.H.G., H.Z., and W.W.D. performed in vivo experiments, including the development of the COH model, optimization and application of the hfCas13X gene editing system, and data analysis. G.Z., S.X., M.J.Z., S.Y.L., R.X.X., Y.C.W., Y.Z., and Y.M. conducted immunohistochemical analyses, collected data on glial activation, and assisted with results presentation. G.Z., Q.X., and S.Y.L. provided RNA‐seq and bioinformatics expertise, developed statistical models, and conducted data analysis. G.Z., Z.L., M.J.Z., H.Z., W.W.D., F.L., Y.C.W, and Y.M. supported in vitro experiments, prepared figures and tables, and contributed to manuscript drafting and revisions. Z.W., G.Z., and Y.M. supervised the entire project, secured funding, guided the research, and actively contributed to manuscript preparation with data contributions from all authors who participated in the project.

## Supporting information



Supporting Information

## Data Availability

The data that support the findings of this study are available from the corresponding author upon reasonable request.
