# Supplementary material for: A High‐Fidelity RNA‐Targeting Cas13X Downregulates Connexin43 in Macroglia: A Novel Neuroprotective Strategy for Glaucoma
Source: Adv Sci (Weinh). 2025 Jun 19;12(33):e15856. doi: 10.1002/advs.202415856 (PMC12412601; doi:10.1002/advs.202415856)
Supplement: Supplementary file 1 — Supporting Information [file ADVS-12-e15856-s001.pdf]

ADVANCED SCIENCE
Open Access

Supporting Information

for *Adv. Sci.*, DOI 10.1002/advs.202415856

A High-Fidelity RNA-Targeting Cas13X Downregulates Connexin43 in Macroglia: A Novel Neuroprotective Strategy for Glaucoma

Guoli Zhao, Zhen Li, Ming-Jie Zhao, Shu-Ying Li, Qing Xia, Shuoyu Xu, Yu Zhang, Yi Wang, Fang Li, Yu-Ling Liu, Yun-Hui Guo, Ruo-Xi Xu, Han Zhou, Hong Zhou, Wen-Wen Ding, Yong-Chen Wang, Yanying Miao* and Zhongfeng Wang**

Supplementary information for

A high-fidelity RNA-targeting Cas13X downregulates connexin43 in macroglia: A novel neuroprotective strategy for glaucoma

Guoli Zhao[1,2,#,*], Zhen Li[1,#], Ming-Jie Zhao[1], Shu-Ying Li[1], Qing Xia[2], Shuoyu Xu[1,2], Yu Zhang[1], Yi Wang[1,2], Fang Li[1], Yu-Ling Liu[2], Yun-Hui Guo[1], Ruo-Xi Xu[1], Han Zhou[1], Hong Zhou[1], Wen-Wen Ding[1], Yong-Chen Wang[3], Yanying Miao[1,*], Zhongfeng Wang[1,*]

[1] State Key Laboratory of Brain Function and Disorders and MOE Frontiers Center for Brain Science, Institutes of Brain Science, Fudan University, Shanghai 200032, China.
[2] Eye Institute and Department of Ophthalmology, Eye & ENT Hospital, Fudan University; Key Laboratory of Myopia and Related Eye Diseases, NHC; Key laboratory of Myopia and Related Eye Diseases, Chinese Academy of Medical Sciences, Shanghai 200032, China
[3] Institute of Neuroscience and Third Affiliated Hospital, Zhengzhou University, Zhengzhou 450052, China.

[#] These authors contributed equally.
*Corresponding author. State Key Laboratory of Medical Neurobiology and MOE Frontiers Center for Brain Science, Institutes of Brain Science, Fudan University, 131 Dongan Road, Shanghai 200032, China. Tel: +86-21-5423-7810; fax: +86-21-5423-7643; E-mails: zfwang@fudan.edu.cn (Z. WANG), glzhao19@fudan.edu.cn (G. ZHAO) and yymiao@fudan.edu.cn (Y. MIAO).

This PDF file includes:

Figs. S1 to S9
Table S1

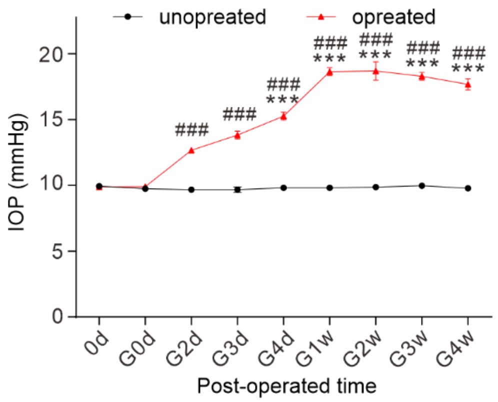

Fig. S1. Changes in IOP in COH mice. The figure shows the changes of IOP with time in the operated eye (indicated by the red triangle) and the contralateral unoperated eye (indicated by the black circle). IOP values were ranging from 9.7 ± 0.1 mmHg to 10.0 ± 0.1 mmHg in the unoperated eye, and they were from 9.9 ± 0.04 mmHg to 18.7 ± 0.7 mmHg in the operated eye. n = 6-107 mice. $^{***}P < 0.001$ vs. 0d. Non-parametric Kruskal-Wallis ANOVA test; $^{\#\#\#}P < 0.001$ *vs*. unoperated eyes at the same time point. Unpaired *t* test with comparisons between each operated eyes and unoperated eyes.

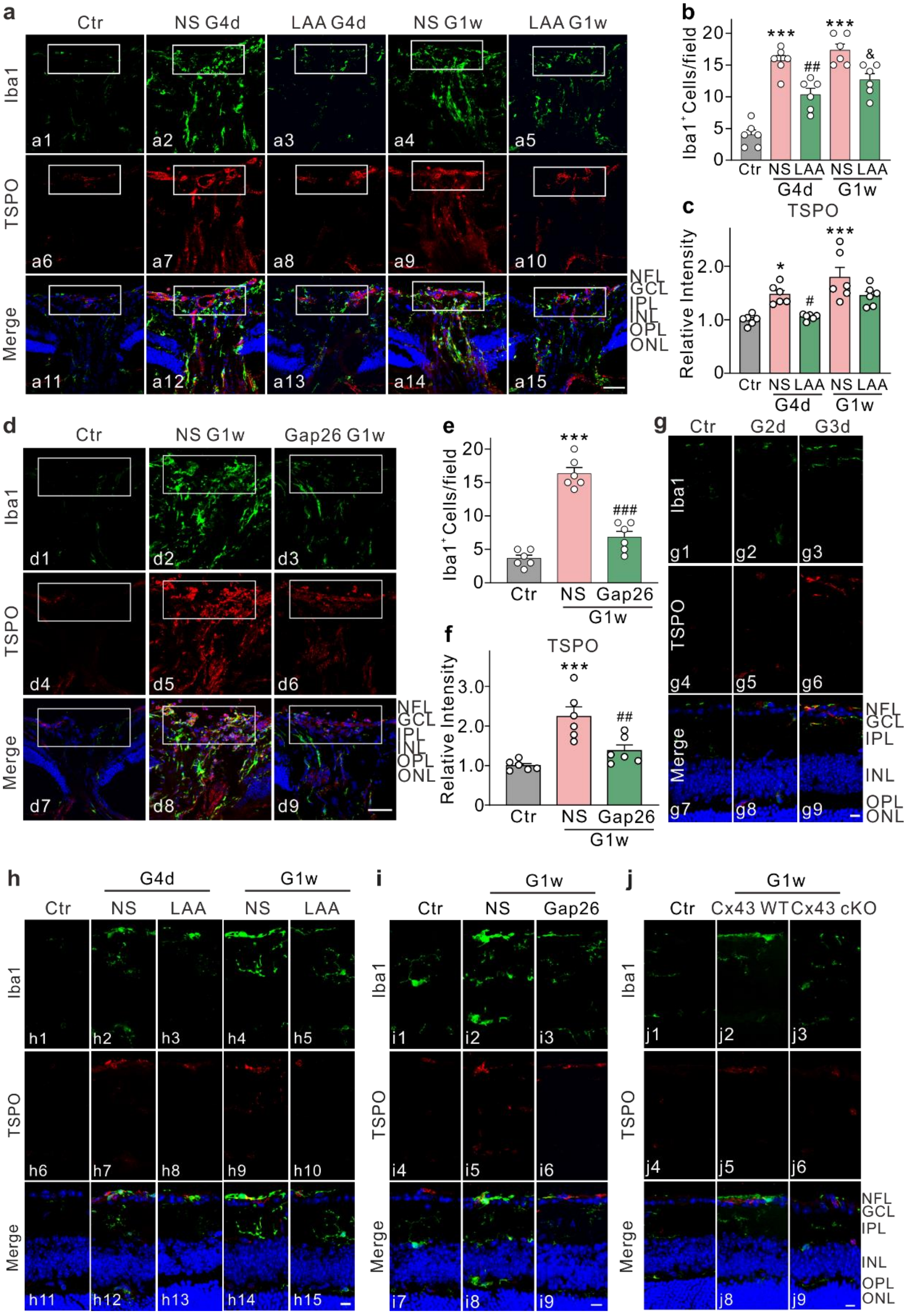

a
Ctr
NS G4d
LAA G4d
NS G1w
LAA G1w
Iba1
TSPO
Merge
a1
a2
a3
a4
a5
a6
a7
a8
a9
a10
a11
a12
a13
a14
a15
NFL
GCL
IPL
INL
OPL
ONL
b
Iba1⁺Cells/field
***
***
##
&
Ctr
NS
LAA
NS
LAA
G4d
G1w
c
TSPO
Relative Intensity
*
#
***
d
Ctr
NS G1w
Gap26 G1w
d1
d2
d3
d4
d5
d6
d7
d8
d9
e
Iba1⁺Cells/field
***
###
Ctr
NS
Gap26
G1w
f
TSPO
Relative Intensity
***
##
g
Ctr
G2d
G3d
g1
g2
g3
g4
g5
g6
g7
g8
g9
h
G4d
G1w
Ctr
NS
LAA
NS
LAA
h1
h2
h3
h4
h5
h6
h7
h8
h9
h10
h11
h12
h13
h14
h15
i
G1w
Ctr
NS
Gap26
i1
i2
i3
i4
i5
i6
i7
i8
i9
j
G1w
Ctr
Cx43 WT
Cx43 cKO
j1
j2
j3
j4
j5
j6
j8
j9

Fig. S2. Effects of CX43-mediated ATP release from astrocytes on microglia. (a) Double immunofluorescence showing the expressions of Iba1 and TSPO in vertical sections of the ONH in the Ctr (a1, a6, a11), normal saline (NS) (a2, a7, a12 and a4, a9, a14) and LAA (a3, a8, a13 and a5, a10, a15) injected groups, respectively. Scale bar: 50 µm for all images. **(b)** Bar chart showing the average number of Iba1-labeled microglia in the white rectangular area in panel **a**. **(c)** Bar chart showing the relative average intensity of TSPO fluorescence signals in the white rectangular area in panel **a**. n = 6 mice. $^{*}P < 0.05$, $^{***}P < 0.001$ *vs*. Ctr; $^{\#}P < 0.05$, $^{\#\#}P < 0.01$ vs. NS G4d group; $^{\&}P < 0.05$ *vs*. NS G1w group. One-way ANOVA with Tukey's multiple comparisons test (for b and c). **(d)** Double immunofluorescence staining of Iba1 (d1, d2, d3) and TSPO (d4, d5, d6) in vertical sections of the ONH in Ctr and COH mice at G1w with NS (NS G1w) or Gap26 (Gap26 G1w) injections. Scale bar: 50 µm for all images. **(e)** Bar chart showing the average number of Iba1-labeled microglia in the white rectangular area in panel **d**. **(f)** Bar chart showing the relative average intensity of TSPO fluorescence signals in the white rectangular area in panel **d**. n = 6 mice. $^{***}P < 0.001$ *vs*. Ctr; $^{\#\#}P < 0.01$, $^{\#\#\#}P < 0.001$ *vs*. NS G1w. One-way ANOVA with Tukey's multiple comparisons test (for e and f). **(g)** Double immunofluorescence staining showing the expression of Iba1 (g1-g3) and TSPO (g4-g6) in retinal vertical sections taken from control (Ctr) and COH mice at G2d and G3d. Scale bar: 10 µm for all images. **(h)** Typical images of the staining of Iba1 and TSPO in retinal vertical sections in Ctr (h1, h6, h11), and COH retinas with NS (h2, h7, h12 and h4, h9, h14) and LAA (h3, h8, h13 and h5, h10, h15) injected groups (at G4d and G1w). Scale bar: 10 µm for all images. **(i)** Double immunofluorescence staining of Iba1 (i1, i2, i3) and TSPO (i4, i5, i6) in retinal vertical section under different conditions. Scale bar: 10 µm for all images. **(j)** The images show double immunofluorescence staining of Iba1 (j1, j2, j3) and TSPO (j4, j5, j6) in retinal

vertical sections in Ctr, Cx43 WT and Cx43 cKO COH mice at G1w. Scale bar: 10 μm for all images.

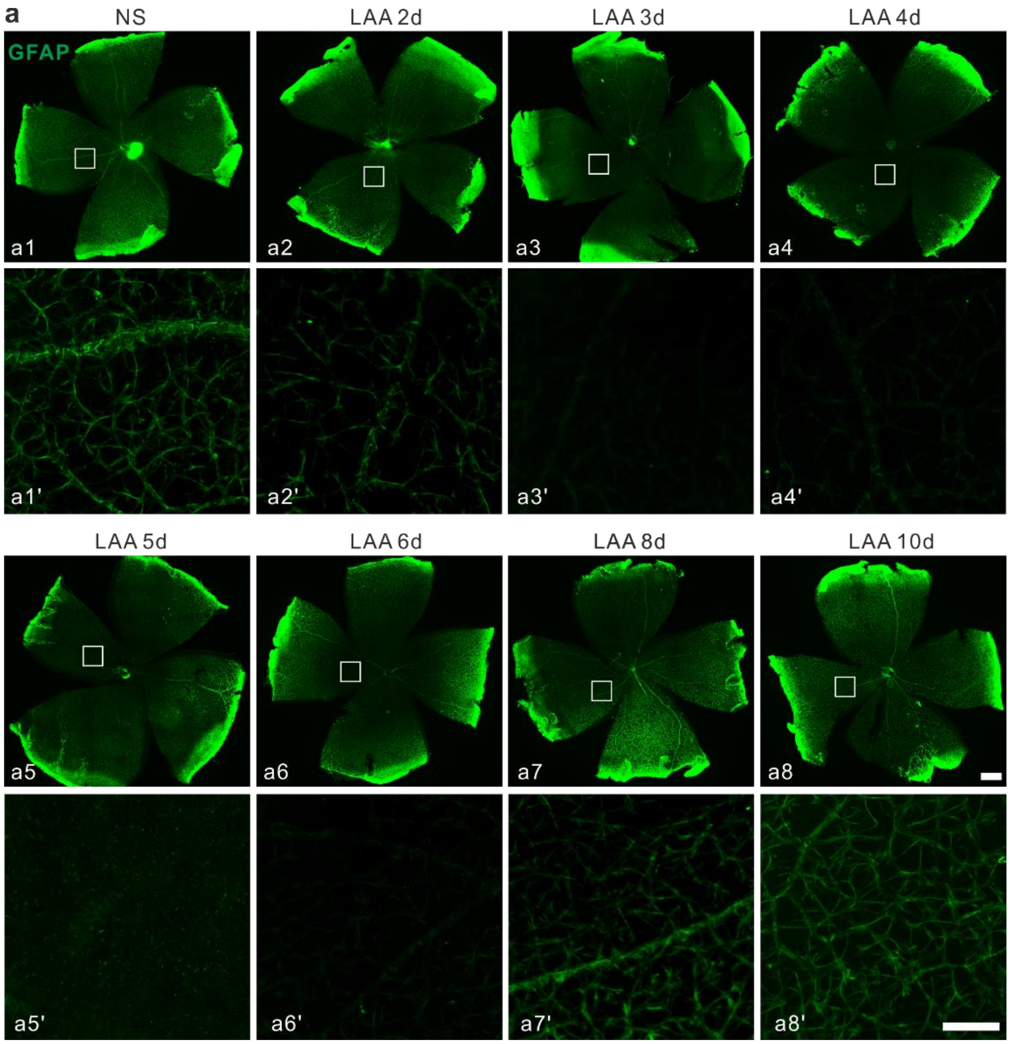

Fig. S3. Evaluation of the efficiency of astrocyte depletion by LAA. (a) Representative images of whole flat-mounted retinas stained for GFAP at 2d, 3d, 4d, 5d, 6d, 8d and 10d (a1-a8) after intravitreal injections of normal saline (NS) and LAA. a1'-a8' are enlarged views of the white rectangular areas of a1-a8, respectively. Scale bar: 50 μm for all images.

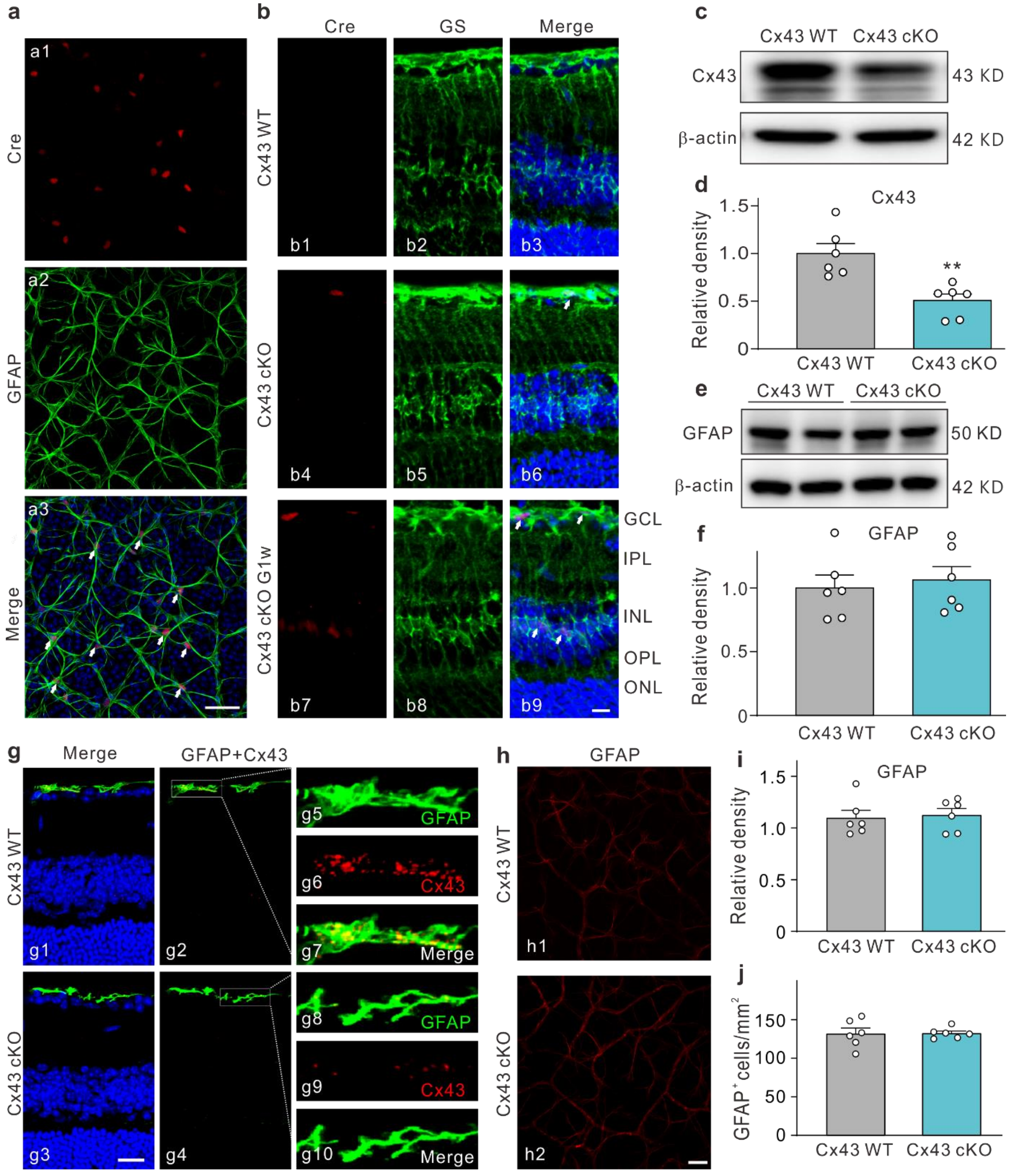

Fig. S4. Evaluation of the efficiency of Cx43 conditional knockout in astrocytes. (a) Representative images showing the double immunofluorescence of Cre (a1) and GFAP (a2) in Cx43 cKO mice. Scale bar: 50 μm for all images. **(b)** Representative images showing the double

immunofluorescence images of Cre (red) and GS (green) staining in Cx43 WT (b1-b3), Cx43 cKO (b4-b6) and Cx43 cKO G1w (b7-b9) retinas, respectively. Scale bar: 10 µm for all images. **(c, d)** Representative immunoblots (**c**) and quantification (**d**) of Cx43 protein in the retina of Cx43 WT and Cx43 cKO mice. n = 6 mice. $^{**}P < 0.01$ *vs*. Cx43 WT. Unpaired *t* test. **(e, f)** Representative immunoblots (**e**) and quantification (**f**) of GFAP protein in the retina of Cx43 WT and Cx43 cKO mice. n = 6 mice. Unpaired *t* test. **(g)** Representative images showing the double immunofluorescence of GFAP and Cx43 in retinal vertical sections of Cx43 WT and Cx43 cKO mice. Scale bar: 20 µm. g5-g7 and g8-g10 are enlarged images of g2 and g4, respectively. **(h)** Representative images of GFAP staining in the retinas of Cx43 WT (h1) and Cx43 cKO (h2) mice. Scale bar: 20 µm. **(i)** Quantification of GFAP fluorescence relative density in the retinas of Cx43 WT and Cx43 cKO mice. **(j)** Quantification of the number of GFAP-positive cells (cells/mm^2) in the retinas of Cx43 WT and Cx43 cKO mice. Unpaired *t* test (for i and j).

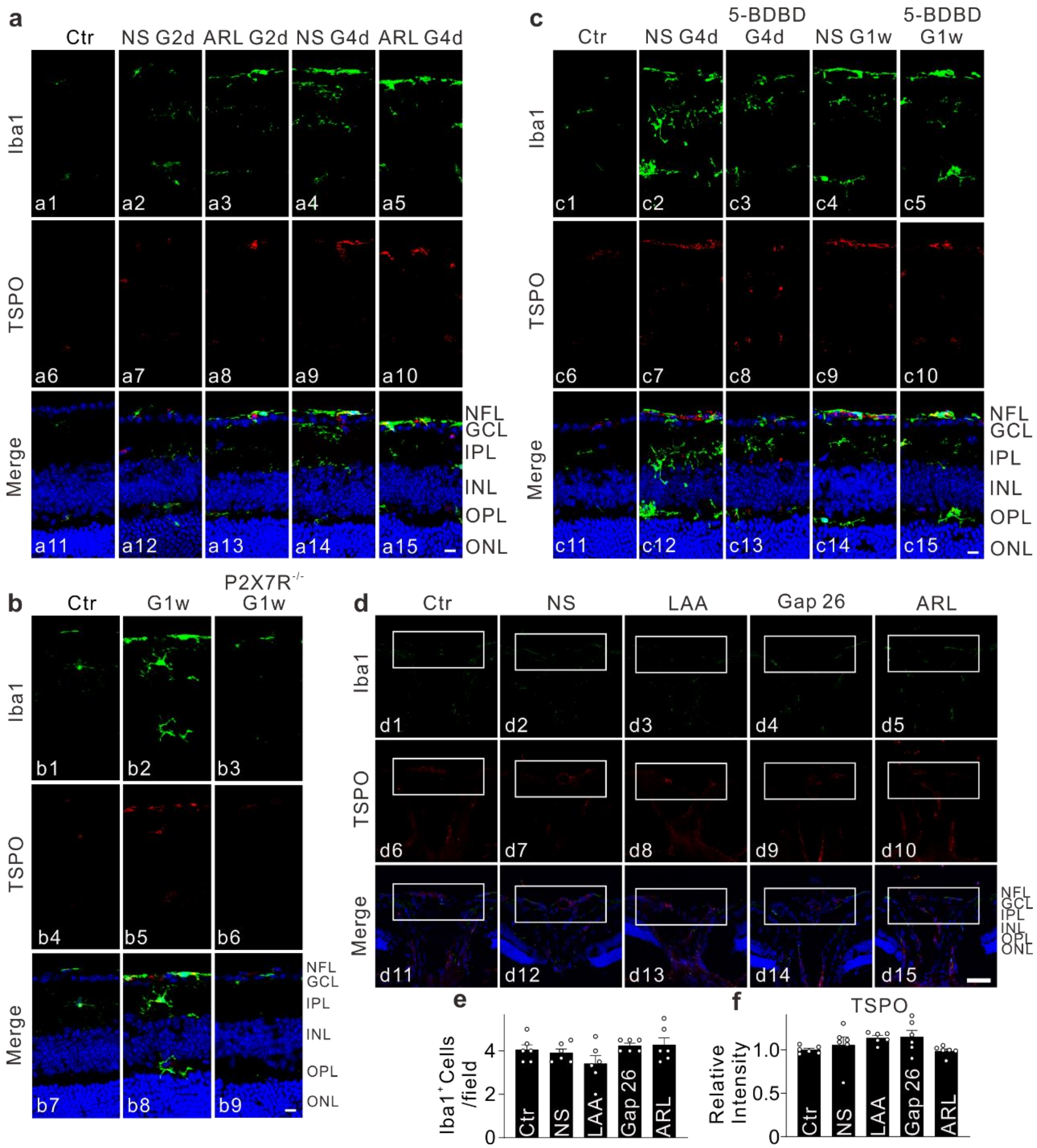

Fig. S5. Effects of P2X7R and P2X4R on microglia activation, proliferation and/or migration in COH mice. (a) Immunofluorescence staining of Iba1 (a1-a5) and TSPO (a6-a10) in retinal vertical sections in Ctr, NS and ARL67156 (ARL) injected COH mice, respectively. **(b)** Immunofluorescence staining of Iba1 (b1-b3) and TSPO (b4-b6) in retinal vertical sections in Ctr, G1w and *P2X7R*$^{-/-}$ G1w mice, respectively. **(c)** Immunofluorescence staining of Iba1 (c1-c5) and TSPO (c6-c10) in retinal vertical sections in Ctr, NS and 5-BDBD injected COH mice at

G4d and G1w, respectively. Scale bar: 10 µm for all images. **(d)** Representative images of Iba1 (green) and TSPO (red) expression in the ONH of Ctr (d1, d6, d11), NS (d2, d7, d12), LAA (d3, d8, d13), Gap 26 (d4, d9, d14), and ARL (d5, d10, d15) injected normal mice, respectively. The merged images (d11-d15) show co-localization of Iba1 and TSPO, with DAPI. Scale bar: 50 µm for all images. **(e)** Bar chart quantifying the average number of Iba1-positive cells per field in the white rectangular areas shown in panel d. n = 6. **(f)** Bar chart quantifying the relative average intensity of TSPO fluorescence signals in the white rectangular areas shown in panel d. n = 6. One-way ANOVA with Tukey's multiple comparisons test (for e and f).

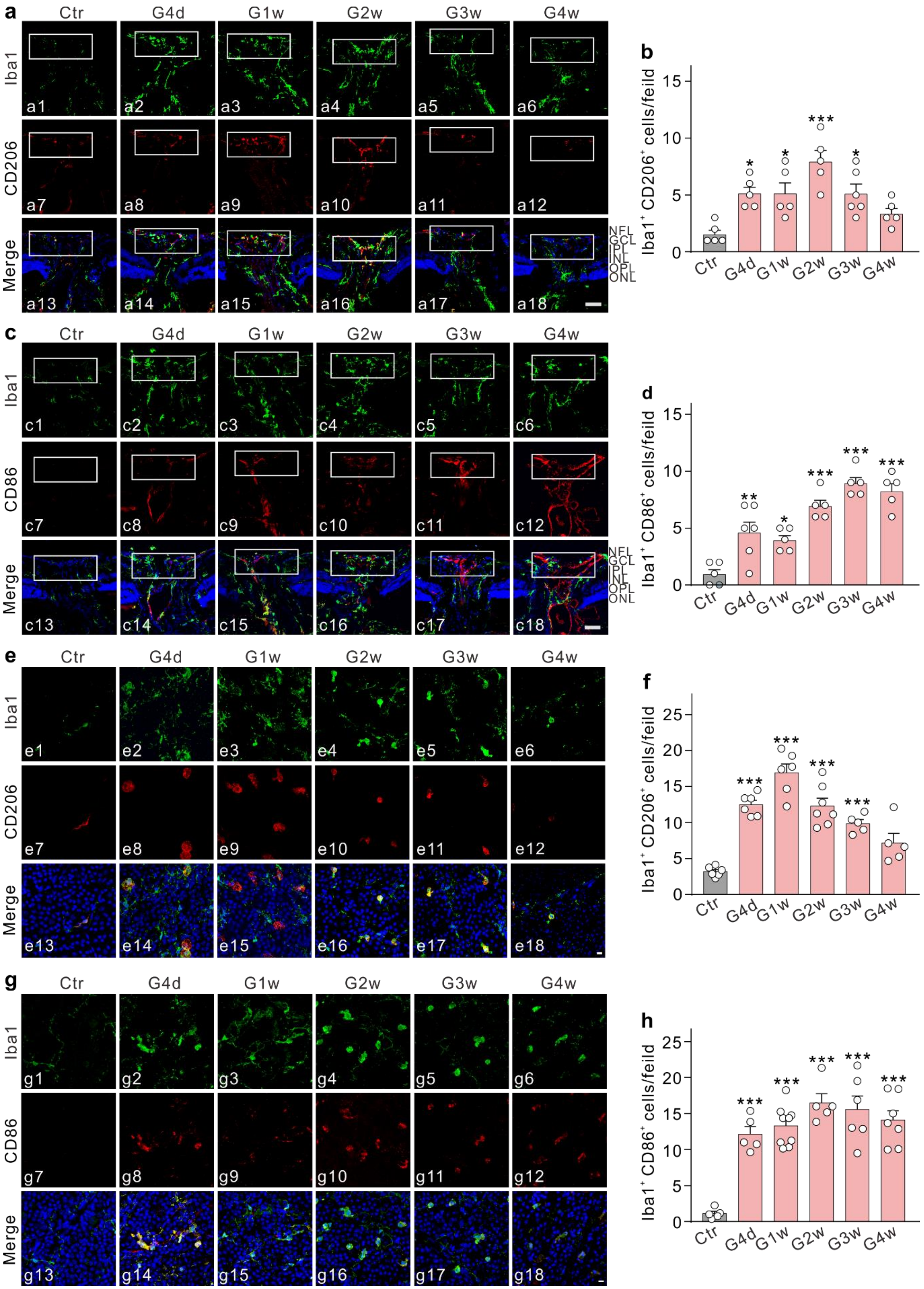
a
Ctr
G4d
G1w
G2w
G3w
G4w
Iba1
CD206
Merge
NFL
GCL
IPL
INL
OPL
ONL
b
Iba1+ CD206+ cells/feild
c
CD86
d
Iba1+ CD86+ cells/feild
e
f
g
h

Fig. S6. The dynamic changes in the number of M1-like and M2-like microglia in COH mice. (a) Representative micrographs of Iba1 (a1-a6) and CD206 (a7-a12) labeled microglia captured from the ONH at different time points of COH mice. Scale bar: 50 μm for all images. **(b)** Quantification of Iba1 and CD206 double labeled cells in the white rectangular area in panel **a**. n = 5-6 mice. $^{*}P < 0.05$, $^{***}P < 0.001$ *vs*. Ctr. One-way ANOVA with Tukey's multiple comparisons test. **(c)** Representative micrographs of Iba1 (c1-c6) and CD86 (c7-c12) labeled microglia captured from the ONH at different time points of COH mice. Scale bar: 50 μm for all images. **(d)** Quantification of Iba1 and CD86 double labeled cells in the white rectangular area in panel **c**. n = 5-6 mice. $^{*}P < 0.05$, $^{**}P < 0.01$, $^{***}P < 0.001$ *vs*. Ctr. One-way ANOVA with Tukey's multiple comparisons test. **(e)** Representative micrographs of Iba1 (e1-e6) and CD206 (e7-e12) labeled microglia captured from whole flat-mounted retinas at different time points of COH mice. Scale bar: 50 μm for all images. **(f)** Quantification of Iba1 and CD206 double labeled cells in each field under different conditions as shown in panel **e**. n = 5-7 mice. $^{***}P < 0.001$ *vs*. Ctr. One-way ANOVA with Tukey's multiple comparisons test. **(g)** Representative micrographs of Iba1 (g1-g6) and CD86 (g7-g12) labeled microglia captured from whole flat-mounted retinas at different time points of COH mice. Scale bar: 50 μm for all images. **(h)** Quantification of Iba1 and CD86 double labeled cells in each field under different conditions as shown in panel **g**. n = 5-9 mice. $^{***}P < 0.001$ *vs*. Ctr. One-way ANOVA with Tukey's multiple comparisons test.

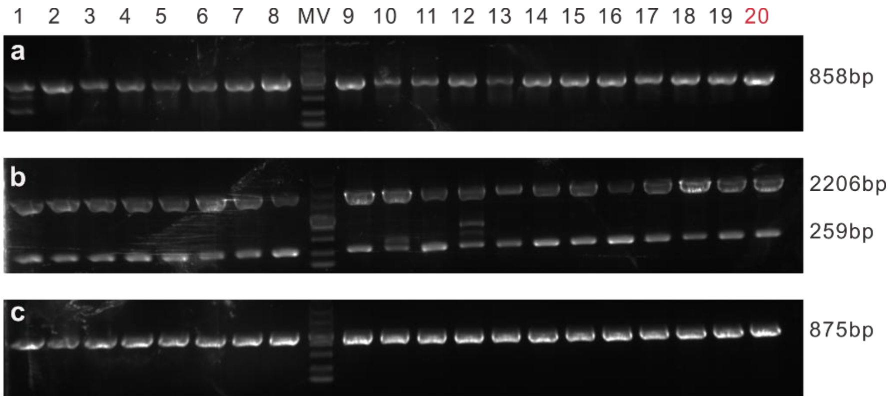

Fig. S7. Identification of N2a stable transfected monoclonal cell lines using nested PCR. Twenty monoclonal N2a cell lines with CMV promoter insertion upstream of the *Gja1* gene were identified using nested PCR, and the results were obtained through gel electrophoresis. **(a)** The gel shows the PCR identification results for the 5' homology arm, with the target band at 858 bp. **(b)** The gel shows the PCR identification results for the inserted CMV promoter fragment, with the target band at 2206 bp. **(c)** The gel shows the PCR identification results for the 3' homology arm, with the target band at 875 bp. Cell lines 1-20 all exhibit high Cx43 expression, and clone 20 was selected for gRNA screening after correct PCR identification and sequencing.

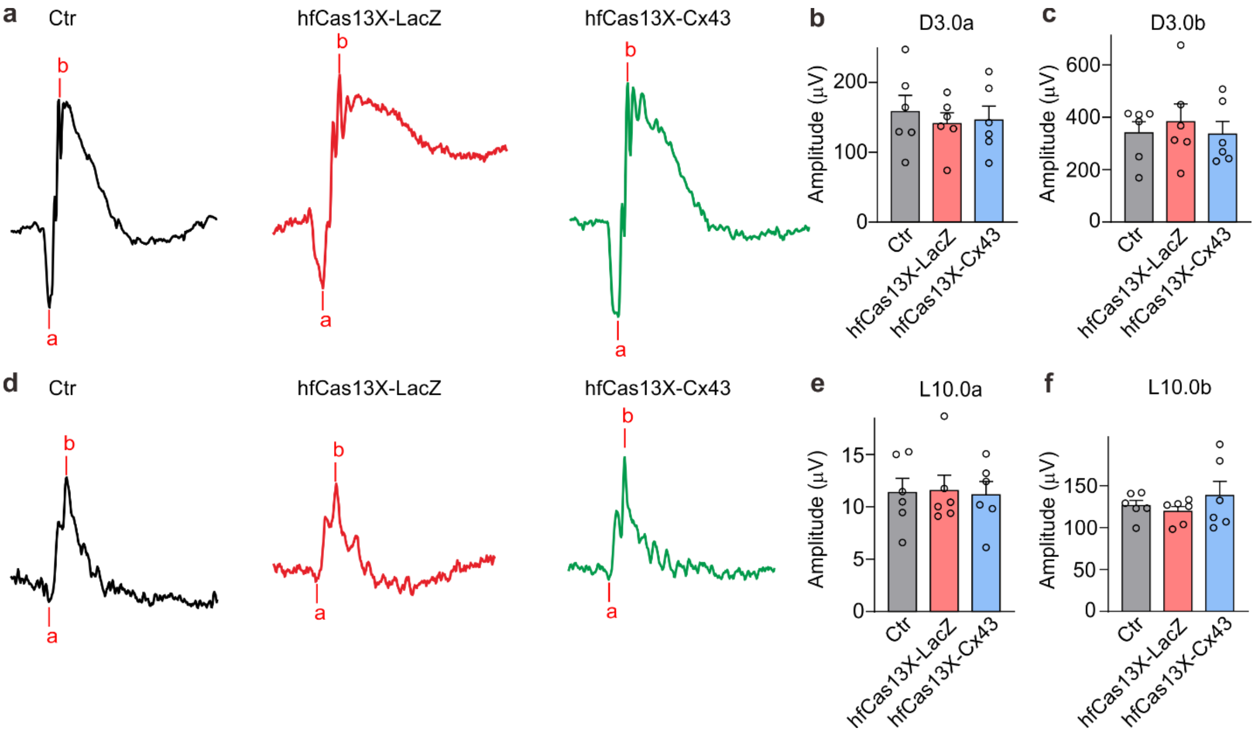

Fig. S8. Injection of hfCas13X-Cx43 virus did not affect retinal photoreceptor function. (a) Dark adaptation 3.0 waveform results of mice injected with hfCas13X-Cx43 virus for 4 weeks. **(b, c)** Bar charts showing the a (**b**) and b (**c**) wave amplitudes under different conditions. n = 6 mice. **(d)** Light adaptation 10.0 waveform results of mice injected with hfCas13X-Cx43 virus for 4 weeks. **(e, f)** Bar charts showing the a (**e**) and b (**f**) wave amplitudes under different conditions. n = 6 mice. One-way ANOVA with Tukey's multiple comparisons test (for b and f). Non-parametric Kruskal-Wallis ANOVA test (for c and e).

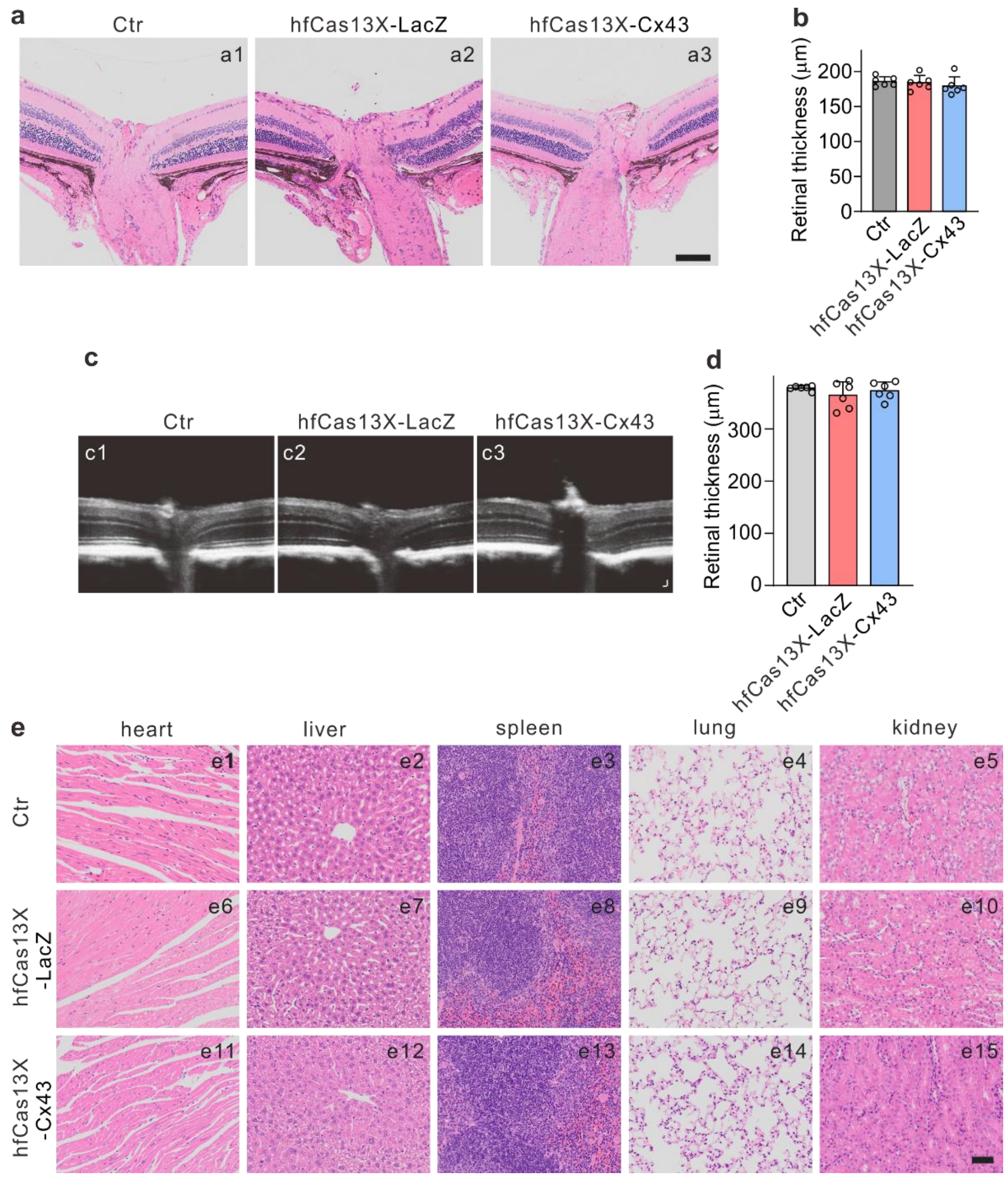

Fig. S9. Injection of hfCas13X-Cx43 virus did not affect retinal structure. (a) Representative HE staining results showing retinal structure in mice with injections of hfCas13X-Cx43 virus at 4 weeks after the injections. Scale bar: 50 μm for all images. **(b)** Statistical map of changes in retinal thickness. n = 6 mice. One-way ANOVA with Tukey's multiple comparisons test. **(c)** Representative OCT results of mice injected with hfCas13X-Cx43 virus for 4 weeks. Scale bar: 50 μm for all images. **(d)** Statistical map of changes in retinal thickness under different conditions. n = 6 mice. One-way ANOVA with Tukey's multiple comparisons test. (e)

Representative HE staining results showing the structures of heart, liver, spleen, lung and kidney in mice with injections of hfCas13X-Cx43 virus at 4 weeks after the injections. Scale bar: 50 µm for all images.

Table S1. SgRNA sequences used in this study.

Targets	guide_sequences
Cx43-sg1	cagcagcttccccaaggcgctccagtcacc
Cx43-sg2	aagagcaccgacagccacaccttccctccg
Cx43-sg3	agacacgaatatgatctgaaggacccagaa
Cx43-sg4	tgaacccatagatgtaccactggatcagca
Cx43-sg5	ctcaatgatattcagagcgagagacaccaa
